# Beyond wound closure: translational opportunities and barriers of mesenchymal stem cells and their extracellular vesicles in burn management

**DOI:** 10.3389/fcell.2026.1816224

**Published:** 2026-05-28

**Authors:** Yuying Wang, Junyan Li, Liyuan Zhang, Juan Liu, Xin Shu, Zhuo Ding, Shangfu Xu

**Affiliations:** 1 Key Laboratory of Cell Engineering, Affiliated Hospital of Zunyi Medical University, Zunyi, China; 2 Guizhou Biomanufacturing Laboratory, Affiliated Hospital of Zunyi Medical University, Zunyi, China; 3 Department of Medical Genetics, Zunyi Medical University, Zunyi, China

**Keywords:** burn immunology, burn wound healing, clinical translation, exosomes, mesenchymal stem cells, vascular regeneration

## Abstract

Burn injury management continues to present multiple clinical challenges, including infection, scar formation, and functional restoration. Mesenchymal stem cells (MSCs) and their secretome (exosomes) have emerged as a cell-free therapy with multitarget potential in inflammation regulation, angiogenesis, epithelial regeneration, and extracellular matrix (ECM) remodeling. Rather than classifying by cell origin, this review is guided by clinical needs and translational pathways to propose a framework for productization and translational development. MSC/EVs strategies exhibit unique advantages along the inflammation-regeneration-fibrosis axis, making them suitable for integration into dressings, delivery systems, and combination therapies. Current obstacles include donor and source consistency, Good Manufacturing Practice (GMP) compliance, standardization of Critical Quality Attributes (CQAs), optimization of administration strategies, and selection of clinically meaningful endpoints. Establishing standardized production and quality-control systems, along with promoting multicenter randomized studies, is essential to achieve high-quality translation from research to clinical practice.

## 1 Introduction

Burn wounds are frequently accompanied by severe inflammation, prolonged healing, and a tendency toward hypertrophic scar formation. As a result, many patients with severe burns require multiple surgeries to prevent and manage acute and long-term complications (Jeschke et al., 2023). Critically, unlike ordinary wounds, burns involve multiple pathophysiological mechanisms, including oxidative stress, excessive inflammatory responses, and impaired epithelial regeneration. In severe burns, elevated oxidative stress further compromises mitochondrial function and triggers a sustained inflammatory response (Buta and Donelan, 2024; Zhang R. et al., 2024). Under normal physiological conditions, the inflammatory response and immune activation are beneficial for recovery (Dobson et al., 2022). After severe burns, however, these responses can become uncontrolled and excessively activated, driving the progression of secondary injury and accounting for the high morbidity and mortality observed in this patient population (Burgess et al., 2022).

Current standards for burn wound care encompass infection prevention, early debridement, dressing changes, and surgical treatment—specifically, autologous skin grafting after excision of necrotic tissue (Anyanwu and Cindass, 2025). Nevertheless, scar formation and crusting remain inevitable consequences of deep burns. By definition, severe burns are characterized as ≥20% TBSA, with full-thickness burns exceeding 5% in adults and ≥10% TBSA in children under 10 years (Jeschke et al., 2020). The current gold standard for treating large-area deep dermal and full-thickness burns is autologous split-thickness skin grafting (STSG): a thin layer of skin containing epidermis and a small portion of dermis, usually expanded using meshing or Meek techniques (Tapking et al., 2024). Despite its widespread adoption, this approach is limited by prolonged preparation and harvesting time, suboptimal functional outcomes, and donor-site morbidity (Asuku et al., 2021). Driven by these persistent limitations, shortening healing time and preventing scar formation remain key priorities in the management of patients with severe burns (Abdul Kareem et al., 2021).

Against this backdrop, innovative cell therapies are rapidly evolving as regenerative strategies for burn wound management and hold great promise (Mirhaj et al., 2022). To date, preclinical and clinical studies have evaluated the roles of various cell types in burn wound treatment, including keratinocytes (KCs), fibroblasts, mesenchymal stem cells (MSCs), embryonic stem cells (ESCs), and induced pluripotent stem cells (iPSCs) (Jo et al., 2021; Shpichka et al., 2019). Of particular interest, studies have found that burned tissue itself can provide viable sources of MSCs. Specifically, MSCs isolated from a patient’s full-thickness skin (burn-derived MSCs, BD-MSCs) have shown promising healing effects in small-sample murine and porcine experiments with no signs of immune rejection (Amini-Nik et al., 2018). Regarding delivery approaches, these cells can be administered via different routes, including local application (e.g., matrix/scaffold-assisted delivery) (Chung et al., 2024) local injections (subcutaneous or intradermal) (Francis et al., 2019), and intravenous systemic delivery (Thompson et al., 2020). Despite these advances, the optimal cell type and delivery method for treating burn wounds have not yet been determined. At the mechanistic level, research indicates that stem cells outperform other cell types in burn wound healing through multiple mechanisms, including reducing granulation tissue formation, enhancing neovascularization, decreasing immune cell infiltration, accelerating extracellular matrix synthesis, inhibiting inflammatory responses, and reducing scar formation and fibrosis, thereby promoting rapid wound healing and effective tissue regeneration (Francis et al., 2019). Focusing on MSCs specifically, as multipotent cells, they can maintain and repair the tissue in which they reside and differentiate into mature cell types such as adipocytes, osteoblasts, and chondrocytes (Maranda et al., 2017). During burn wound healing, MSCs secrete a range of cytokines according to wound site requirements, including proliferation-promoting, anti-inflammatory, and anti-fibrotic factors delivered via exosomes. Through this targeted secretory modulation, MSCs support faster healing and reduced scarring (Lei et al., 2025; Zhang et al., 2023a). In parallel, soluble factors secreted by MSCs recruit and enhance the activity of resident cells in the target tissue, thereby reinforcing endogenous mechanisms of tissue repair and regeneration (Ramos-Gonzalez et al., 2023). A critical advantage of the EV-based approach is that, compared with MSC therapy, MSC-derived exosomes are more stable under various pathophysiological conditions, exhibit lower immunogenicity (Wang et al., 2025a), and carry no cell-therapy-related risks (Ye et al., 2025).

**FIGURE 1:**
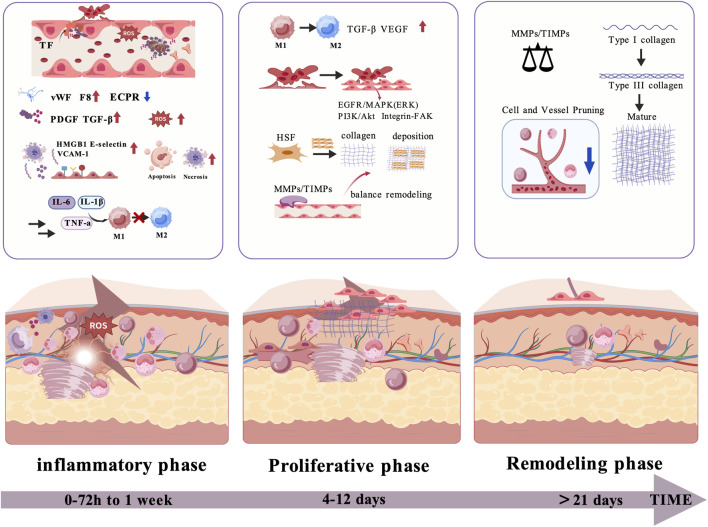
The dynamic progression of burn wound healing: From acute inflammation to tissue remodeling. This schematic illustrates the three continuous and overlapping phases of skin repair following a burn injury. Acute Inflammatory Phase (0–72 h to 1 week): Thermal insult triggers vascular endothelial damage and Tissue Factor (TF) exposure, initiating the coagulation cascade. Necrotic tissue liberates Damage-Associated Molecular Patterns (DAMPs, e.g., HMGB1), activating the complement system and driving a “cytokine storm” (IL-6, IL-1β, TNF-α). Endothelial cells express adhesion molecules (e.g., VCAM-1) to facilitate leukocyte infiltration. Proliferative Phase (4–12 days): The focus shifts to matrix reconstitution and barrier restoration. Macrophages transition from a pro-inflammatory M1 phenotype to a pro-regenerative M2 phenotype, secreting TGF-β and VEGF to stimulate angiogenesis. Human Skin Fibroblasts (HSFs) proliferate and migrate, driven by EGFR/MAPK and PI3K-Akt signaling, to initiate robust collagen deposition. Remodeling Phase (>21 days): Inflammation subsides and nascent vasculature undergoes pruning. Type III collagen is progressively replaced by Type I collagen. The Extracellular Matrix (ECM) undergoes extensive reorganization governed by the balance between Matrix Metalloproteinases (MMPs) and their inhibitors (TIMPs), culminating in a mature scar. TF, Tissue Factor; vWF, von Willebrand Factor 8; F8, Coagulation Factor III; ECPR, Endothelial Protein C Receptor; HMGB1, High Mobility Group Box 1; VCAM-1, Vascular Cell Adhesion Molecule-1; TGF-β, Transforming Growth Factor-β; EGFR, Epidermal Growth Factor Receptor; MAPK, Mitogen-Activated Protein Kinase; ERK, Extracellular signal-Regulated Kinase; MMPs, Matrix Metalloproteinases; TIMPs, Tissue Inhibitors of Metalloproteinases; TNF-α, Tumor Necrosis Factor-α; IL-6, Interleukin-6; IL-12, Interleukin-12; IL-1β, Interleukin-1β; ROS, Reactive Oxygen Species; HSF, Human Skin Fibroblasts; VEGF, Vascular Endothelial Growth Factor.

In this review, we design a clinically problem-centered “MSCs and their EVs application roadmap” that examines the burn course to identify key intervention points where MSCs and EVs can act, and propose targeted strategic breakthroughs at these critical junctures. Building on this framework, we provide perspectives on clinical translation, including product form, delivery platforms, trial design, limitations, and evidence gaps, with the aim of offering a structured framework for the clinical application of MSC-based therapies for burns and supporting the development of new technologies (Figure 1).

## 2 Burn pathophysiology: critical nodes in MSC/EVs vesicle-mediated intervention

### 2.1 A three-dimensional perspective on burn pathophysiology

Burn injury does not represent a discrete, localized event confined to a single time point but rather a dynamic process that evolves concurrently along temporal, histological, and systemic axes. Consistent with this multidimensional perspective, burn wound healing is conventionally stratified into the acute inflammatory phase, the proliferative phase, and the remodeling phase, each underpinned by distinct pathophysiological mechanisms (Peña and Martin, 2024).

During the acute inflammatory phase, which typically spans the first 0–72 h and the first week post-injury, thermal injury induces vascular endothelial damage and tissue factor (TF) exposure. This precipitates rapid activation of the coagulation cascade, during which platelets undergo adhesion, activation, and aggregation to form the initial hemostatic plug (Gando et al., 2024). Concurrently, the endothelium loses key endogenous anticoagulant mechanisms—including thrombomodulin and endothelial protein C receptor (EPCR)—and shifts toward a procoagulant phenotype (Iba et al., 2026). Within venous sinusoids and related vascular compartments, von Willebrand factor (vWF) and coagulation factor VIII (FVIII) are markedly upregulated, further potentiating platelet adhesion and thrombin generation (Zhou et al., 2024). Activated platelets simultaneously release platelet-derived growth factor (PDGF), TGF-β, and a spectrum of chemokines, thereby establishing an inflammatory priming milieu (Lihao et al., 2024; Chen L. et al., 2025). Within hours, necrotic tissue liberates substantial quantities of damage-associated molecular patterns (DAMPs), including high mobility group box 1 protein (HMGB1) and DNA fragments (Scaffidi et al., 2002). These DAMPs engage pattern recognition receptors and activate the complement system (Yang et al., 2022; Gavriilaki et al., 2021), driving pronounced elevations in pro-inflammatory cytokines such as TNF-α, IL-6, and IL-1β. In turn, these mediators directly induce endothelial expression of E-selectin, vascular cell adhesion molecule-1 (VCAM-1), and chemokines (De Oliveira et al., 2016), facilitating leukocyte rolling, firm adhesion, and transendothelial migration of neutrophils and monocytes into the injured tissue (Obeagu, 2025). Beyond these immediate vascular and inflammatory events, extensive cellular apoptosis and necrosis occur at the wound site. Importantly, these are attributable not only to direct thermal destruction but also to secondary mechanisms including ischemia-reperfusion injury, neuroendocrine stress responses, and fluid-electrolyte dysregulation (Chen R. et al., 2025). A critical pathological hallmark is that the glycolysis-driven M1 macrophage phenotype fails to transition toward the oxidative phosphorylation (OXPHOS)-dependent M2 phenotype. Consequently, M1 macrophages sustain secretion of pro-inflammatory cytokines, including IL-1β, IL-6, and TNF-α, which amplify local inflammatory signaling and promote further monocyte recruitment, thereby establishing a self-perpetuating positive feedback loop (Mulder et al., 2024). Compounding this inflammatory dysregulation, oxidative stress is markedly exacerbated: mast cell-derived histamine augments xanthine oxidase-mediated reactive oxygen species (ROS) generation. In turn, excessive ROS production in turn drives systemic inflammatory response syndrome (SIRS), immune dysregulation, heightened susceptibility to infection and sepsis, progressive tissue injury, and multiple organ dysfunction (Parihar et al., 2008). During the acute phase, moist wound surfaces with abundant exudate substantially elevate infection risk (Sarkar et al., 2025), while systemic complications may include distributive shock and inadequate end-organ perfusion (Hundeshagen et al., 2024). This phase is therefore critical for limiting injury propagation and protecting remote organ function.

Following pathogen clearance and wound debridement, the tissue enters the proliferative phase, which is characterized by resolution of the inflammatory milieu and transition into an active repair program. Also termed the subacute or proliferative phase, this period typically spans days 4–21 post-injury (Childs and Murthy, 2017) and is defined by granulation tissue formation. During this phase, the primary wound-healing objective shifts from “damage control and pathogen elimination” toward “matrix reconstitution, barrier restoration, and perfusion recovery.” At the cellular level, locally elaborated reparative signals progressively predominate, and the phenotypic transition of macrophages from M1 toward M2/pro-regenerative states represents a pivotal inflection point. Subsequently, human skin fibroblasts (HSFs) undergo rapid proliferation within the injured zone and initiate robust collagen synthesis (Lenselink, 2015). Simultaneously, epithelial cells migrate and proliferate to resurface the wound, thereby preventing infection and transepidermal water loss (Pastar et al., 2014). Mechanistically, driven by EGFR/MAPK (ERK) and PI3K-Akt signaling, keratinocytes adhere to and migrate across newly exposed extracellular matrix components, including fibronectin and collagen fragments. Integrin-FAK pathway activation coordinates the coupled “adhesion–migration–proliferation” axis (Mcbride et al., 2021; Cuba et al., 2026). In parallel, endothelial cells within the wound bed organize into nascent capillary networks, progressively restoring tissue perfusion (Diomede et al., 2020). In the mid-proliferative phase, ongoing collagen synthesis is accompanied by deposition and architectural remodeling. This process is governed by the finely balanced activity of matrix metalloproteinases (MMPs) and their tissue inhibitors (TIMPs), which regulate matrix degradation and reorganization and thereby provide the structural scaffold requisite for functional tissue restoration (Mayorca-Guiliani et al., 2025). In the late proliferative phase, fibroblasts and myofibroblasts synthesize and deposit ECM proteins, culminating in scar tissue formation (Nourian Dehkordi et al., 2019). Of particular therapeutic relevance, this phase represents a state in which residual inflammation and active repair coexist, offering a therapeutic window amenable to MSC/EVs-mediated modulation of the immune microenvironment, promotion of angiogenesis, and facilitation of wound closure.

Subsequently, the tissue enters the remodeling phase. During this phase, the inflammatory response progressively abates, immune cell populations diminish, the nascent vasculature undergoes pruning, and the ECM undergoes extensive reorganization, ultimately yielding a rigid scar (Gushiken et al., 2021). At the matrix level, fibroblasts primarily crosslink and remodel the provisional ECM deposited during earlier phases. A defining biochemical feature is that type III collagen is progressively replaced by type I collagen, consolidating and reinforcing the ECM architecture over time and resulting in the formation of a mature, mechanically robust scar (Hinz, 2007).

### 2.2 Unresolved clinical challenges in burn management

The management of burn injuries presents several persistent clinical challenges that remain incompletely resolved. These encompass four principal domains: (1) the control of infection and excessive inflammation, (2) the promotion of rapid and high-quality re-epithelialization and angiogenesis, (3) the mitigation of hypertrophic scarring and contracture, and (4) the systemic regulation of burn-associated inflammation and immune dysregulation in patients with large total body surface area (TBSA) burns.

The first challenge concerns infection and hyperinflammation. Burn wounds are inherently susceptible to bacterial colonization and infection. Bacterial infection not only impedes wound healing but may also precipitate systemic inflammatory response syndrome (SIRS), which can culminate in dysregulated inflammatory cascades and ultimately progress to multiple organ dysfunction syndrome (MODS) (Ye et al., 2023). Accordingly, the rational management of infection and hyperinflammation, along with the prevention of secondary infectious complications, remains a critical and unresolved challenge in burn care.

The second challenge pertains to re-epithelialization and angiogenesis. Re-epithelialization, defined as the coverage of the wound surface by a newly generated epidermal layer, represents the initial and indispensable step toward restoration of skin integrity and barrier function. This process advances centripetally from wound margins, with keratinocytes adhering to extracellular matrix (ECM) proteins and reorganizing into a continuous, stratified, differentiating neoepidermis (Laigle et al., 2026). Within this domain, in the context of extensive cutaneous defects, the relative proportions of epidermal stem cells (EpSCs) and dendritic epidermal T cells (DETCs) critically determine the extent of re-epithelialization. However, the precise mechanisms by which DETCs modulate EpSC behavior to regulate this process remain to be fully elucidated (Liu et al., 2022). Turning to angiogenesis, this process plays a central role in wound healing by supplying nascent regenerating tissue with oxygen and nutrients and is of particular importance in ischemic and chronic wounds (Yan et al., 2025). Robust neovascularization constitutes the biological foundation for restoring tissue viability and sustaining adequate perfusion. Importantly, as two cardinal processes in burn wound healing, effective re-epithelialization and angiogenesis are intimately interrelated: rapid and complete re-epithelialization minimizes wound exposure and thereby reduces infection risk (Laigle et al., 2026; Lee et al., 2023). Critically, these processes exhibit phase-specific regulatory requirements: during the inflammatory phase, it is necessary to attenuate endothelial inflammatory responses and limit excessive immune cell infiltration, whereas during the proliferative phase, dysregulated angiogenesis must be constrained within physiological bounds (Bogale et al., 2025). Therefore, effective strategies for concurrently promoting both processes and achieving high-quality, functionally competent wound healing across these distinct phases warrant further investigation.

The third challenge involves hypertrophic scarring and wound contracture. These represent frequent and clinically significant sequelae of burn injury, arising from aberrant fibroblast activation during the wound healing process. Hypertrophic scars are histopathologically characterized by pathological fibroblast activation, manifested by upregulated expression of Kv3.1 channels and excessive ECM deposition (Seo et al., 2021). Mechanistically, evidence indicates that increased matrix stiffness drives fibroblast-to-myofibroblast transdifferentiation, generating a self-amplifying “stiffness–activation” positive feedback loop (Ezzo et al., 2024; Walker et al., 2021). Regarding current therapeutic limitations, autologous split-thickness skin grafting (STSG) remains the standard of care for deep burns, yet it is frequently associated with post-grafting contracture and dyspigmentation (Chen K. et al., 2022). Similarly, although glucocorticoid therapy is widely employed, studies utilizing 11β-HSD1 knockout mouse models have demonstrated that glucocorticoid signaling, while accelerating wound closure, may concurrently exacerbate collagen deposition and tissue fibrosis (Tsai et al., 2023). Furthermore, although certain naturally derived ECM-based biomaterials demonstrate capacity to facilitate wound healing, their efficacy in suppressing scar contracture remains limited (Chen L. et al., 2022). Taken together, therapeutic options in this domain remain inadequate, underscoring the urgent need for novel conceptual frameworks and treatment strategies.

**FIGURE 2:**
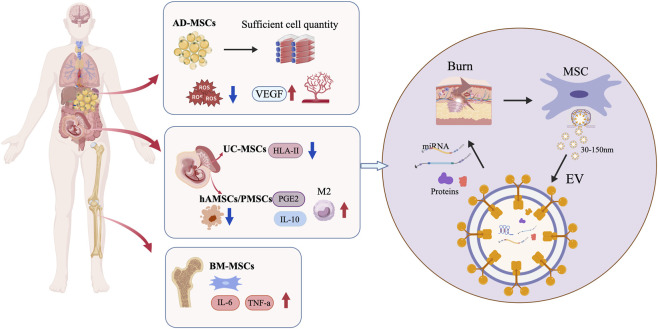
MSCs/sEVs sources and their partial roles in burn injuries. This schematic illustrates the heterogeneity of MSC sources and their specialized paracrine roles in mitigating burn-induced injury. Tissue-Specific Sources: BM-MSCs and AD-MSCs: Traditional sources from bone marrow and adipose tissue, known for their robust pro-angiogenic signaling (via VEGF) and modulation of acute inflammatory mediators (e.g., TNF-α). UC-MSCs, PMSCs and hAMSCs: Perinatal-derived MSCs (Umbilical Cord, Placenta, and Human Amniotic Membrane). Notably, hAMSCs and PMSCs offer superior immunomodulatory properties and lower immunogenicity. They secrete potent anti-inflammatory cytokines (e.g., IL-10) and hepatocyte growth factor (HGF), effectively quenching the “cytokine storm” associated with severe burns. The Cargo Delivery: The figure depicts the biogenesis of EVs as cargo carriers that encapsulate specific miRNAs, proteins, and lipids. These cell-free agents overcome the limitations of live-cell therapy, such as low survival in the burn microenvironment and potential tumorigenicity. VEGF, Vascular Endothelial Growth Factor; HLA-II, Human Leukocyte Antigen Class II; PGE2, Polyethylene Glycol 2000; IL-10, Interleukin-10; IL-6, Interleukin-6; TNF-α, Tumor Necrosis Factor-α.

The fourth challenge, complementary to these local complications, is systemic inflammation and immune dysregulation. Patients sustaining large TBSA burns frequently develop pronounced systemic inflammation and immune dysregulation. Emerging evidence indicates that dysregulation of both myeloid and lymphoid immune cell compartments following burn injury correlates significantly with disease severity and mortality. Of particular note, this pattern of immune dysregulation is broadly observed across acute respiratory distress syndrome (ARDS), traumatic injury, and burn patients, suggesting the existence of conserved pathological mechanisms of immune dysfunction shared across critical illness states (Moore et al., 2025). Therefore, developing effective strategies for systemic immunomodulation—capable of counteracting burn-induced systemic inflammation and immune dysregulation-represents a pivotal direction for improving outcomes in patients with major burns (Figure 2).

## 3 MSCs and their derived products

### 3.1 Definition and comparative overview of major MSC sources

In the most recent identification criteria published by the International Society for Cell and Gene Therapy (ISCT) in 2025 (Renesme et al., 2025), MSCs are formally designated as “Mesenchymal Stromal Cells,” with emphasis placed on their functional mechanisms as being primarily mediated through the secretion of cytokines and extracellular vesicles (EVs) to modulate the local microenvironment. Of particular relevance to burn therapy, this conceptual shift carries significant implications, given that the cytokine storm and oxidative stress induced by severe burns critically necessitate this form of active paracrine intervention. Under this updated framework, the criteria require provision of stemness evidence when invoking the term “stem cell” and place heightened emphasis on potency assessment and critical quality attributes (CQAs), thereby establishing a more rigorous framework for clinical translation in burn medicine.

Owing to their low immunogenicity and potent immunomodulatory capacity, MSCs can precisely regulate the phenotypic switching of immune effector cells—including T lymphocytes and macrophages—through direct cell-to-cell contact or paracrine signaling, thereby counteracting the pro-inflammatory microenvironment characteristic of the acute burn phase. Based on tissue of origin, MSCs are broadly categorized into bone marrow-derived MSCs (BM-MSCs), adipose-derived MSCs (AD-MSCs), umbilical cord-derived MSCs (UC-MSCs), and amniotic membrane- and placenta-derived MSCs (hAMSCs/PMSCs), among others.

As the most extensively studied MSC subtype, BM-MSCs have been shown to promote granulation tissue formation during angiogenesis by expressing high levels of FGF and VEGF, thereby facilitating neovascularization (Revilla et al., 2024). Importantly, this finding has also been validated in a porcine model of severe radiation burns (Linard et al., 2018), extending its relevance beyond thermal injury to radiation-induced cutaneous damage. In a rat model of second-degree burns, BM-MSCs significantly downregulate wound healing mediators, including transforming growth factor-β (TGF-β), interleukin-6 (IL-6), tumor necrosis factor-α (TNF-α), matrix metalloproteinase-9 (MMP-9), and microRNA-21. Concurrently, the expression of heat shock protein-90α (HSP-90α) is significantly upregulated, thereby restoring normal skin structure and reducing scar formation (Abdel-Gawad et al., 2021).

However, their invasive procurement procedure and age-dependent decline in cell yield impose practical limitations for emergency application in large TBSA burns. Addressing these limitations, AD-MSCs can be isolated with exceptional efficiency: 300 mL of adipose tissue yields a sufficient cellular reservoir (Asch et al., 2025), providing a reliable cell manufacturing source for burn patients. Within the burn wound microenvironment, the pronounced antioxidant capacity of AD-MSCs enables effective ROS scavenging, thereby attenuating secondary oxidative injury following thermal insult. In parallel, their high-level vascular endothelial growth factor (VEGF) secretion promotes neovascularization in ischemic wound zones (Deng et al., 2025; Wu Y. et al., 2021). Turning to UC-MSCs, these cells are characterized by exceptionally low immunogenicity, attributable to diminished HLA class II expression, and robust proliferative capacity (Chang et al., 2021), rendering them well-suited as off-the-shelf therapeutic products for severe burns (>30% TBSA). In a mouse burn experiment, UC-MSCs were injected via the tail vein at 0, 1, and 3 h post-burn. Notably, the UC-MSC group demonstrated preserved blood-brain barrier (BBB) integrity after burns, an effect attributed to reduced IL-6 and IL-1β levels and increased Major Facilitator Superfamily Domain-Containing Protein 2A (MFSD2A) levels (Yang et al., 2020). Derived from perinatal tissues, hAMSCs/PMSCs exhibit the lowest immunogenicity among MSC subtypes and possess distinctive clinical value (Allouh et al., 2025; Datta et al., 2021; Kim et al., 2025). Mechanistically, hAMSCs effectively suppress heat stress-induced cellular apoptosis *in vitro*, whereas PMSCs directly polarize macrophages toward the M2 phenotype through secretion of prostaglandin E2 (PGE2), IL-10, and related immunomodulatory factors. Collectively, these properties demonstrate considerable therapeutic potential in promoting full-thickness tissue regeneration and suppressing hypertrophic scar formation.

### 3.2 MSC-derived extracellular vesicles

According to the Minimal Information for Studies of Extracellular Vesicles (MISEV2023) guidelines published by the International Society for Extracellular Vesicles (ISEV) in 2023, the term “exosome” in its strict sense refers to endosome-derived vesicles with a diameter of 30–150 nm. However, given that current mainstream isolation methodologies, including differential ultracentrifugation and size-exclusion chromatography, cannot reliably discriminate between vesicles of endosomal origin and those generated by plasma membrane budding, the ISEV recommends preferential adoption of the operationally defined term “small extracellular vesicles (small EVs; sEVs).” Consistent with this consensus, throughout this article, the unified terminology of “MSC-derived small extracellular vesicles (MSC-sEVs)” or “extracellular vesicles (EVs)” is employed.

In the context of clinical translation, the choice between cell-based therapy and cell-free approaches requires careful consideration of their respective biological characteristics relative to the burn pathological environment. A comparative analysis across three key dimensions—mechanism of action, safety and immunogenicity, and standardization and logistics—reveals distinct profiles for each approach. Regarding mechanism of action, MSCs possess inherent “environmental sensing” capabilities, enabling dynamic modulation of their secretory profile in response to wound-specific stimuli such as hypoxia and inflammatory cytokine gradients. By contrast, MSC-sEVs function as a “static concentrated cargo,” directly regulating target cells through encapsulated miRNAs, proteins, and lipids in a manner that is more precisely controlled but lacks autonomous environmental responsiveness (Mellado et al., 2026; Izquierdo-Altarejos et al., 2024). n terms of safety and immunogenicity, MSCs are subject to donor variability and frequently encounter constraints in clinical application (Van Hoecke et al., 2025). Conversely, MSC-sEVs exhibit minimal immunogenicity, are incapable of autonomous replication, and thereby circumvent risks of tumorigenicity and vascular embolism (Ren et al., 2026). Regarding standardization and logistics, MSCs require stringent cold-chain management to preserve viability, with batch-to-batch consistency markedly affected by donor heterogeneity and passage number (Mcbride et al., 2021; Cuba et al., 2026). By contrast, MSC-sEVs possess greater physicochemical stability, can be stored long-term at −80 °C, and are more amenable to scalable, standardized manufacturing and ready-to-use clinical deployment (Lei et al., 2025; Tieu et al., 2020).

Beyond these comparative considerations, it is essential to examine the specific therapeutic effects of MSC-sEVs in the burn context. Burn wounds are characteristically associated with severe inflammatory responses, and MSC-sEVs have been shown to attenuate excessive inflammation through modulation of immune effector cell function. As an example, adipose-derived MSC-sEVs (AD-MSC-sEVs) exert pronounced anti-inflammatory effects, dampening the inflammatory cascade within the burn microenvironment and thereby creating favorable conditions for subsequent tissue repair (Wang et al., 2025b; Yeh et al., 2025).

Regarding angiogenesis, MSC-sEVs carry pro-angiogenic factors and key regulatory microRNAs—including 28 functionally critical miRNAs identified in recent studies—which effectively activate endothelial cell function and facilitate neovascularization (Lei et al., 2025; Chen Y. et al., 2025). Turning to scar management, hypertrophic scar overgrowth represents a principal long-term sequela of burn wound healing. During the scar remodeling phase, MSC-sEVs further contribute to tissue repair and regeneration through multiple complementary mechanisms: (1) promoting endothelial cell proliferation and migration while facilitating ECM remodeling within the dermis to improve tissue repair (Zhang H. et al., 2024) and attenuate photoaging. (2) delivering anti-apoptotic signals to enhance survival of cells in damaged tissue (Zhang et al., 2020). (3) modulating gene expression in target cells to regulate cellular proliferation and differentiation, thereby accelerating the repair of injured tissue (Zhang et al., 2021). and (4) scavenging free radicals to reduce oxidative damage and retard cellular senescence (Zhang et al., 2023b). Taken together, MSC-sEVs are emerging as a novel and highly promising cell-free therapeutic platform, with growing clinical investigation across a broad spectrum of disease indications.

## 4 Therapeutic intervention I: acute inflammatory phase-immune and inflammatory reprogramming

### 4.1 Limitations of conventional anti-inflammatory strategies

Conventional anti-inflammatory strategies employed in burn management demonstrate significant limitations in both their application and therapeutic efficacy. Systemic glucocorticoids, despite their potent anti-inflammatory properties, are associated with immunosuppression, elevated infection risk, delayed wound healing, and potential systemic metabolic adverse effects when administered at high doses or over prolonged durations. Evidence indicates that long-term or high-dose glucocorticoid use induces dermal thinning and impairs wound closure, whereas locally generated glucocorticoids, activated via 11β-hydroxysteroid dehydrogenase type 1 (11β-HSD1), may modulate scar formation. Systemic administration, however, risks exacerbating tissue injury (Tsai et al., 2023). Compounding this concern, glucocorticoids may excessively suppress inflammatory responses, thereby compromising the function of immune effector cells such as M2 macrophages that are indispensable for wound repair (Xiang et al., 2025). Conventional topical formulations, including topical corticosteroids and non-steroidal anti-inflammatory drugs (NSAIDs), exhibit poor dermal penetration due to the skin barrier effect, resulting in limited therapeutic efficacy and potential systemic side effects (Barati et al., 2024). Although topical anti-inflammatory agents can reduce local inflammatory burden, they may concurrently impede re-epithelialization, induce local skin fragility, and predispose to secondary infection. A further limitation is that most topical agents are mechanistically narrow-spectrum, targeting discrete inflammatory mediators such as IL-6 or TGF-β1, and are therefore incapable of comprehensively modulating the complex inflammatory network that emerges following burn injury (Mulder et al., 2024). Driven by these collective limitations, there has been growing clinical interest in MSC/EVs-based immunomodulatory and inflammatory reprogramming strategies, with the expectation that multi-targeted, systemic regulation may achieve safer, more controllable, and higher-quality wound healing outcomes.

### 4.2 Immunomodulatory effects of MSC/EVs in burn injury

When evaluating the existing mechanistic evidence, we categorize the data into burn-specific model studies and studies using similar (non-burn-specific) models. Critically, these two categories exhibit inherently different levels of applicability when inferring therapeutic use in burn settings.

#### 4.2.1 Burn-specific preclinical studies

In burn-specific model evidence, the main advantage of MSCs lies in their ability to synergistically mitigate inflammation through multiple immunomodulatory mechanisms. As an example, in a skin burn study, AD-MSCs can modulate the expression of Th cells and IL-10, thereby upregulating relevant chemokines and cytokines to suppress inflammatory cascades and promote resolution of inflammation (Abdul Kareem et al., 2021). In a complementary finding, UC-MSCs have been shown to inhibit inflammatory cell infiltration, reduce IL-6 and TNF-α levels, and simultaneously enhance IL-10 and TSG-6, ultimately accelerating burn wound healing (Nourian Dehkordi et al., 2019). Extending this evidence to placental-derived MSCs, PL-MSCs have been demonstrated to exhibit immunosuppressive molecular programs. Specifically, they may express high levels of immune checkpoint ligands PD-L1 and PD-L2, which interfere with T-cell proliferation-associated processes (e.g., cell-cycle progression), thereby limiting the inflammatory amplification mediated by T cells (Shojaei et al., 2019).

Importantly, the immunosuppressive phenotype of MSCs is not static. Upon exposure to pro-inflammatory cytokines such as IFN-γ, TNF-α, IL-1α, and IL-1β, MSCs are further activated they then express chemokines and induce inducible nitric oxide synthase (iNOS). Collectively, these immunoregulatory effectors suppress the responsiveness of T cells to inflammatory stimuli, thereby enabling more comprehensive regulation of the inflammatory response (Maxson et al., 2012; Hu et al., 2018).

#### 4.2.2 Studies using similar models

In studies using similar models, the evidence mainly manifests in three aspects: (1) the excessive shift of M1 macrophages toward an M2 phenotype, (2) overproduction of neutrophil extracellular traps (NETs), and (3) endothelial activation with increased permeability. Within the burn-related immune landscape, it is essential to recognize that macrophage polarization is not a simple, one-directional process of suppressing or promoting inflammation. Rather, it represents a continuous spectrum and dynamic balance between M1 and M2 states. MSCs and their EVs are considered to promote a shift toward an M2-like phenotype through multiple signaling pathways, while theoretically preserving an initial defensive inflammatory response that is beneficial for debridement and anti-infection.

The signaling pathways frequently cited in existing literature vary considerably in their evidence strength and hierarchies. Regarding the Wnt/β-catenin pathway, ADSC-Exos can inhibit TNF-α and IL-6 to induce M2 polarization, thereby activating Wnt/β-catenin signaling and promoting keratinocyte proliferation and epithelial formation. However, this evidence is derived from general full-thickness skin defect models (Wang et al., 2025b), and its direct applicability to burn-specific inflammation remains to be established. Concerning the AKT/FoxO1 pathway, in an *in vitro* model in which macrophages are stimulated with LPS, TGF-β secreted by MSCs may promote M2-like polarization via the AKT/FoxO1 pathway and improve phagocytic capacity (Liu et al., 2019). Yet this conclusion is based on an *in vitro*, non-skin mechanism study, making the extrapolation to immunomodulation in burn wounds comparatively speculative. Turning to NF-κB inhibition and PPAR-γ/STAT6 activation, MSC-sEVs cargo—including miRNAs, proteins, and lipid molecules, has been reported to suppress the NF-κB pro-inflammatory pathway and activate PPAR-γ/STAT6 transcriptional programs, thereby promoting the expression of M2 markers such as Arg1 and Ym1 (Dong et al., 2021; Xue et al., 2024). However, these data are derived from facial nerve injury models and are associated with local and systemic inflammatory responses. Of critical note, all the above mechanisms lack burn-specific clinical mechanistic evidence. To date, no clinical studies have verified the efficiency of M1-M2 polarization in burn patients using mechanistic endpoints.

Regarding neutrophil extracellular traps (NETs), burn injury is often accompanied by excessive NETs release, which leads to tissue damage and an imbalance in host defense against infection. Stratifying the evidence for this effect reveals three tiers of varying proximity to the burn context.

The first tier, derived from a non-cutaneous organ injury model (hepatic ischemia-reperfusion), Lu et al. demonstrated that UCMSC-sEVs suppress NET formation by transferring functional mitochondria to hepatic neutrophils (Lu et al., 2022). This constitutes indirect circumstantial evidence of mechanistic plausibility, albeit from a tissue context that diverges substantially from burn pathophysiology. The second tier, from an adjacent injury model (acute ocular chemical burn), reported that hAMSC-conditioned medium reduces NET release and promotes corneal wound healing (Navas et al., 2018). This bears some analogical relevance to thermal burn injury, although differences in tissue origin and injury mechanism persist, limiting direct extrapolation. The third tier, drawn from a non-burn chronic wound model (diabetic wounds), showed that MSC-conditioned medium attenuates excessive NET formation through anti-inflammatory and antioxidant properties (Lin et al., 2026). This represents supportive but more distal evidence, further removed from the acute burn microenvironment. Taken together, these studies suggest at a mechanistic level that MSC-sEVs possess the capacity to modulate NET formation. However, neither the model selection nor the available direct burn evidence is sufficient to support burn-specific conclusions—underscoring the need for dedicated burn-specific NET investigations.

Regarding endothelial activation and increased permeability, burn-induced endothelial activation and permeability enhancement are key processes leading to local edema and impaired perfusion. Research has shown that MSC-sEVs enhance endothelial barrier integrity through downregulation of TGF-β and Wnt/β-catenin signaling and upregulation of tight junction proteins (occludin, claudin-5, and VE-cadherin). Critically, these findings are derived in part from burn or scald animal models, constituting relatively direct supporting evidenc (Elakkawi et al., 2026). By contrast, evidence for UCMSC-sEVs-mediated inhibition of endothelial-to-mesenchymal transition (EndMT) is primarily derived from non-burn fibrosis or vascular injury models (Zhao Y. et al., 2023). As a result, its role in post-burn fibrosis and vascular leakage remains a mechanistic analogy awaiting validation in burn-specific experimental systems.

In n summary, within the mechanistic network of burn inflammatory reprogramming, NF-κB pathway suppression and PPAR-γ/STAT6 activation likely occupy upstream regulatory positions, converging through M2 macrophage polarization as a shared downstream effector node. Nevertheless, the finding that MSC-sEVs from distinct cellular origins activate the same downstream phenotypic outcome (M2 polarization) via divergent pathways—including STAT3, Wnt/β-catenin, and PTEN/AKT—raises two competing interpretations. On one hand, this convergence may suggest the existence of functional redundancy among these pathways. On the other hand, it may reflect source-specific mechanistic differences. Critically, this question has yet to be addressed through systematic comparative investigation in burn-specific models, representing a fundamental knowledge gap that must be resolved to inform rational source selection for clinical translation.

## 5 Therapeutic intervention II: proliferative phase, promoting high-quality re-epithelialization and angiogenesis

### 5.1 Multi-targeted effects of MSCs/exosomes on “high-quality skin regeneration”

Although existing regenerative skin substitutes have achieved notable advances in wound coverage, they remain substantially deficient in restoring dermal architecture, appendageal regeneration, and long-term functional and aesthetic outcomes. Contemporary constructs can approximate the multilayered structure of native skin, encompassing the epidermis, dermis, and subcutaneous adipose layer, yet they remain unable to recapitulate the coordinated and rapid regeneration of vascular networks, peripheral nerves, and skin appendages (Hao et al., 2026; Mazio et al., 2024). Dermal substitutes serve as scaffolding templates for deep cutaneous defect repair, but rapid vascularization continues to be a principal barrier to their clinical performance (Weng et al., 2022). Murine skin equivalents containing hair follicles, sebaceous glands, and eccrine sweat glands have been established *in vitro*. However, the regeneration of human skin appendages continues to face formidable challenges (Hosseini et al., 2022). Furthermore, current skin substitutes are unable to fully replicate the chemical, mechanical, and biological properties of native skin. For instance, three-dimensionally bioprinted skin constructs can modulate Young’s modulus within the range of 87–213 kPa through structural adjustment, yet they require further optimization to match the biomechanical characteristics of human skin (Yu K. et al., 2023).

n contrast, MSCs and their secreted extracellular vesicles offer a multi-targeted immunomodulatory and regenerative strategy capable of promoting “high-quality” skin regeneration across multiple biological hierarchies.

#### 5.1.1 Burn-specific preclinical studies

Among burn-specific model evidence, the main aspects can be categorized into angiogenesis, dermal healing, epithelial barrier reconstruction, and metabolic reprogramming.

Regarding angiogenesis, MSC-sEVs do not simply induce *de novo* vessel formation but rather promote functional neovascularization through a constellation of complementary mechanisms. These include facilitating endothelial cell recruitment, secreting insulin-like growth factor 1 (IGF-1) to stimulate endothelial progenitor cell proliferation and reinforce vascular barrier integrity (Hosseini et al., 2022), secreting fibroblast growth factor (FGF) (Yu K. et al., 2023) and VEGF/TGF-β (Zhu et al., 2024), and activating the PTEN/AKT/VEGF signaling axis to potentiate angiogenesis (Wang et al., 2025c). This refined, multi-mechanistic regulation, distinct from non-selective pro-angiogenic stimulation, reduces the proportion of leaky, structurally immature capillaries and improves overall tissue perfusion efficiency.

Building on this concept, the latest evidence indicates that AD-MSC-sEVs exhibit substantial potential in regenerative medicine and have been shown to be beneficial for wound repair, including burn injuries. Specifically, studies have found that 3D-printed microfibrillar cultures (3D-EVs) exert stronger pro-angiogenic effects, suggesting that an HA-loaded, releasable 3D exosome repair system based on AD-MSCs can significantly improve therapeutic outcomes for wound healing (Zhu et al., 2024). In a complementary line of investigation, hypoxic preconditioning has been shown to markedly enhance the capacity of MSCs to secrete EVs. By enriching pro-angiogenic cargoes such as miR-125a-5p, these EVs demonstrate stronger endothelial protection within the burn-specific hypoxic pathological microenvironment (Wang et al., 2025d). Extending these findings to large-animal models, a study using miniature Bama pigs with deep partial-thickness burns demonstrated that treatment with different doses of UC-MSCs (from 1 × 10^6^ to 1 × 10^8^ cells) resulted in increased microvascular density in the high-dose group. The expression levels of angiogenesis-related factors, including VEGF and ANG-2, were upregulated, indicating that UC-MSCs promote wound neovascularization (Liu L. et al., 2024). Of particular translational significance, this study was among the first to demonstrate in a large-animal model that higher doses of UC-MSCs can synergistically accelerate wound healing through multiple pathways, encompassing both inflammation inhibition and angiogenesis promotion, thereby providing key evidence supporting the clinical translational potential of stem-cell therapy for burns. More recently, a similar investigation employed MSCs derived from human induced pluripotent stem cells (iPSCs) to treat a Yorkshire pig full-thickness burn model. The treatment significantly reduced the expression of pro-inflammatory factors (e.g., IL-1β and TNF-α) and modulated the expression of matrix metalloproteinases (MMP-2 and MMP-9), thereby promoting angiogenesis and tissue remodeling and creating a favorable microenvironment for wound healing (Farahat et al., 2025).

Beyond angiogenesis, high-quality dermal healing depends on the formation and activity of human skin fibroblasts (HSFs) and keratinocytes (KCs). Regarding regulation of the dermal microenvironment, a study of second-degree burns demonstrated that hypoxic preconditioning of MSCs effectively interferes with the conversion of HSFs into myofibroblasts. Mechanistically, this effect mainly involves increasing the secretion of hepatocyte growth factor (HGF) and suppressing the TGF-β/Smad signaling axis (Elakkawi et al., 2026; He et al., 2022). Consistent with this paradigm, in the context of acute radiation-induced dermatitis, ADSC-derived exosomes promote cutaneous regeneration through modulation of the TGF-β/Smad2/3 pathway (Li M. et al., 2025).

Turning to epithelial barrier reconstruction, MSC-sEVs deliver functional biologically active molecules such as microRNAs, thereby regulating keratinocyte (KC) migration, proliferation, and differentiation. As an illustrative example, in a mouse skin wound model, AD-MSC-sEVs improved the function of oxidative stress-injured HaCaT cells via miR-10b delivery and promoted re-epithelialization (Liao et al., 2022). At a systems level, single-cell RNA sequencing studies reveal that human umbilical cord MSC-sEVs can remodel intercellular communication networks within the wound microenvironment, indirectly supporting the structural and functional integrity of the epidermal-dermal interface (Liu Y. et al., 2024).

Finally, in the domain of metabolic reprogramming, MSC-sEVs have been demonstrated to modulate immune cell polarization, including promotion of M2 macrophage transition, whose metabolic signature is conducive to inflammatory resolution and tissue repair support (Geng et al., 2023; Li W. et al., 2025).

#### 5.1.2 Similarity model research

In studies using similar models, the relevant findings can be divided into three main aspects: angiogenesis, metabolic reprogramming, and regeneration of accessory organs as a related outcome.

At the signaling pathway level, the Notch signaling pathway is recognized as one of the key axes in angiogenesis and tissue repair. Together with pathways such as PI3K/Akt/mTOR and Wnt/β-catenin, it forms a therapeutic target network (Chen C. et al., 2023). Consistent with this framework, multiple studies indicate that the therapeutic effects of MSC-sEVs partially depend on the modulation of pathways such as Notch, thereby preventing excessive angiogenesis or structural disorganization (Tan et al., 2026; Chaurasia et al., 2022). Regarding metabolic reprogramming, a study using a renal fibrosis model showed that MSC-sEVs can alleviate oxidative stress-induced cellular injury and apoptosis by delivering antioxidant-related factors, such as modulating components of pathways including Nrf2 and NOX4. Through this mechanism, MSC-sEVs indirectly improve energy utilization efficiency (Liu et al., 2025a).

Regarding potential leads for skin appendage regeneration, animal studies suggest that signals associated with sweat gland, hair follicle, and peripheral nerve regeneration may be activated or reprogrammed through local microenvironmental modulation at the injury site. Among the most promising leads, hUCMSC-sEVs have been shown to attenuate dihydrotestosterone (DHT)-induced disruption of the hair follicle growth cycle by suppressing NF-κB activation, thereby reducing local inflammation and restoring follicular structural integrity (Yu et al., 2025; Kang et al., 2024), while leveraging miR-1827 to optimize intercellular communication and maintain hair follicle cycling (Shrestha et al., 2025; Chen J. et al., 2023). Concerning sweat gland regeneration, although direct evidence remains limited, the Wnt/β-catenin signaling axis—which governs folliculogenesis—is considered to harbor potential for reprogramming toward sweat gland regeneration, given the developmental homology between the hair follicle and sweat gland unit (Nagano et al., 2024).

It must be acknowledged that current research evidence is predominantly derived from animal models. Consequently, the translation of these discrete mechanistic breakthroughs into a coherent, system-level regenerative paradigm applicable to the human context, given species-specific differences in skin appendage distribution and the complexity of the systemic inflammatory response in burns, remains a critical translational evidence gap.

### 5.2 Envisioned applications toward surgical workflow reconstruction

Building upon available *in vitro* and animal experimental evidence, this article proposes the integration of EVs as auxiliary bioactive carriers embedded within the surgical workflow to reconstruct the burn wound treatment pathway, achieving localized delivery in conjunction with autologous or allogeneic skin grafts and engineered dermal substitutes. At the mechanistic level, the role of EVs in modulating inflammatory responses is well established. Specifically, MSC-sEVs regulate the balance between pro- and anti-inflammatory mediators, creating a local microenvironment conducive to tissue repair at the wound site (Suzdaltseva et al., 2022). This immunomodulatory capacity is pivotal for suppressing excessive early wound inflammation and preventing healing impairment attributable to chronic inflammatory persistence (Marofi et al., 2021). Beyond immunomodulation, EVs also demonstrate considerable promise in promoting epithelial regeneration and angiogenesis. Evidence indicates that MSC-sEVs stimulate neovascularization, which is particularly critical for wound perfusion re-establishment and tissue viability (Wu R. et al., 2021; Zhang et al., 2023c).In parallel, MSCs have been shown to possess epithelial differentiation potential, particularly in gingival-derived MSCs where *in vitro* differentiation toward epidermal-like cells has been observed, suggesting that EVs may carry cognate signaling molecules capable of promoting re-epithelialization (Li Y. et al., 2024). Complementing these effects, MSC-sEVs have been demonstrated to enhance fibroblast activity, collagen deposition, and matrix remodeling, thereby optimizing the structural repair process of the wound bed (Bai et al., 2020).

Regarding delivery strategies, the combination of intraoperative spray application and postoperative controlled-release dressing systems aligns with current requirements for efficient localized delivery and sustained bioactivity of exosomes. Notably, the literature acknowledges that, although MSC-sEVs are broadly recognized for their wound healing benefits, ensuring their effective release and retention at the wound site remains a critical challenge for clinical translation (Chen X. et al., 2025). To address this challenge, two prospective conceptual frameworks are proposed for comparative consideration. Concept A, designated “early single-stage surgery with biological modulation,” involves simultaneous skin grafting and MSC-sEVs administration within a single operative procedure. This enables immediate intervention at the peak of inflammation and initiation of the regenerative program, thereby shortening healing duration and minimizing infection risk. Concept B, designated “EV-assisted iterative grafting strategy,” is oriented toward large TBSA burn wounds and emphasizes continuous biological modulation to improve the local microenvironment, enhance graft take rates, and reduce scar formation. Supporting both approaches, existing evidence indicates that MSC-sEVs not only facilitate wound healing but also improve scar quality and attenuate fibrosis (Sun et al., 2025), providing a mechanistic rationale for multi-intervention approaches.

**FIGURE 3:**
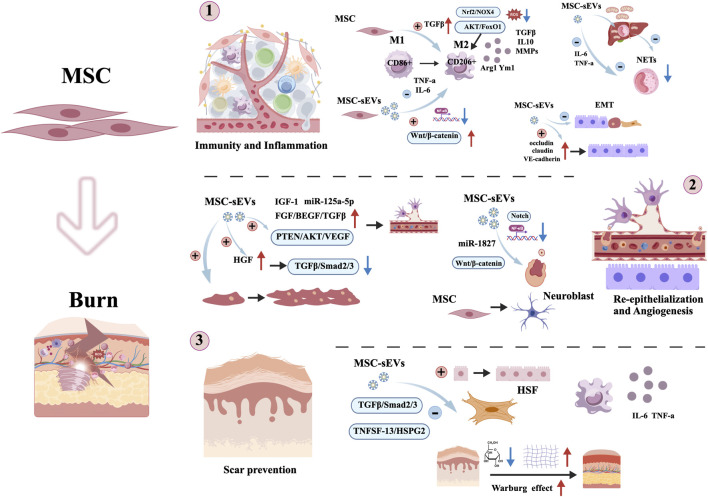
The role of MSC/EVs in different stages of burn disease progression. This schematic illustrates the biological functions by the clinical timeline of burn recovery: Part I: Acute Inflammatory Phase—Immunomodulatory Mechanisms (Section 4.2): Upper panel. Focusing on the early post-burn period where MSC-EVs mitigate the “cytokine storm.” The primary mechanism involves orchestrating M2 macrophage polarization and suppressing pro-inflammatory cascades (e.g., TNF-α, IL-6) and neutrophil overactivation. Part II: Proliferative Phase—Burn-Specific Preclinical Studies and Analogous Models (Section 5.1): Middle panel. Highlighting how engineered EVs (via hypoxia or IFN-γ priming) accelerate wound closure by promoting re-epithelialization, angiogenesis, and fibroblast activation. This section integrates evidence from burn-specific and analogous skin regeneration models to optimize EV therapeutic efficacy. Part III: Remodeling Phase—Preclinical Evidence and Translational Barriers (Section 6.1): Lower panel. Focusing on long-term repair quality and anti-fibrotic interventions. It discusses the regulation of ECM deposition by EVs to prevent hypertrophic scarring. MSC, Mesenchymal Stem Cell; MSC-Exos, MSC-exosomes; TGFβ, Transforming Growth Factor-β; Arg1, Arginase-1; Ym1, Chitinase-Like Protein 3; TNF-α, Tumor Necrosis Factor-α; IL-6, Interleukin-6; IL-10, Interleukin-10; MMPs, Matrix Metalloproteinases; Nrf2, Nuclear Factor Erythroid 2-Related Factor 2; NOX4, NADPH Oxidase 4; FoxO1, Forkhead Box Protein O1; NETs, Neutrophil Extracellular Traps; EMT, Epithelial-Mesenchymal Transition; HGF, Hepatocyte Growth Factor; IGF-1, Insulin-like Growth Factor-1; FGF, Fibroblast Growth Factor; BEGF, Brain-Derived Endothelial Growth Factor; PTEN, Phosphatase and Tensin Homolog; AKT, Serine/Threonine Kinase/Protein Kinase B; Smad2/3, Mothers against decapentaplegic homolog 2/3; TNFSF-13, Tumor Necrosis Factor Superfamily Member 13; HSPG2, Heparan Sulfate Proteoglycan 2; HSF, Human Skin Fibroblasts.

At present,multi-targeted angiogenesis and regeneration evidence is currently predominantly derived from *in vitro* systems or small animal models. Critically, evidence for skin appendage regeneration—encompassing sweat gland and hair follicle restoration—remains the weakest domain, with virtually no human-derived data available. To facilitate translational advancement, study design should prospectively address EV source characterization and quality control, optimal administration timing and release kinetics, carrier biomaterial compatibility, sample size calculation and statistical design, and ethical and regulatory requirements. Ultimately, systematic evaluation should be conducted through rigorously controlled comparative studies to generate the high-quality evidence needed to guide clinical implementation (Figure 3).

## 6 Therapeutic intervention III: remodeling phase-long-term scar management

### 6.1 Role of MSCs/exosomes in fibrosis prevention

Burn scar formation is a complex pathophysiological process that has traditionally been regarded as a static endpoint following wound closure. Driven by this conceptual framework, prevailing clinical strategies have tended toward reactive intervention, employing pressure garments, intralesional glucocorticoid injections, or laser ablation only after established scar hypertrophy (Harris et al., 2024). However, these modalities are frequently constrained by poor patient adherence, high recurrence rates, and an inability to reverse deep tissue contracture.

#### 6.1.1 Burn-specific preclinical studies

In the mid-to-late stages of scar formation, MSC-sEVs exhibit potential reversal capabilities. Although the exact pathogenesis of pathological scars remains incompletely understood, excessive activity of local fibroblasts (HSFs) is recognized as a fundamental driver of both wound healing and scar formation (Wilkinson and Hardman, 2020). Building on this premise, studies have demonstrated that UC-MSC-sEVs can effectively regulate HSFs, vascular endothelial cells, and macrophages, thereby inhibiting myofibroblast-mediated fibrosis and promoting wound healing while concurrently suppressing scar formation in mouse burn models (Yang et al., 2024).

#### 6.1.2 Similarity model research

During the early post-traumatic inflammatory resolution phase, MSC-sEVs suppress excessive fibrogenesis through multidimensional mechanisms. The most canonical among these involves the TGF-β/Smad signaling axis. Specifically, evidence demonstrates that MSC-sEVs downregulate TGF-β1 expression and block Smad2/3 phosphorylation, thereby inhibiting pathological HSF hyperactivation (Zhang G. et al., 2024; Lee et al., 2022). Extending these findings to *in vivo* models, MSC-sEVs have been shown to attenuate peritoneal and cutaneous fibrosis progression by reducing inflammatory infiltration and improving microcirculatory perfusion (Yu F. et al., 2023). Of particular clinical relevance, this early-stage interception is of critical importance in large TBSA burns, where sustained inflammatory cascades typically constitute the principal driver of aberrant fibrogenesis. At the cellular signaling level, studies have further demonstrated that MSC/EVs can alleviate scar formation through pathway-specific mechanisms. As an example, Zhang et al. (2023d) reported that BM-MSC-sEVs alleviate hypertrophic scars by inhibiting HSFs through the TNFSF-13/HSPG2 signaling pathway. In a parallel line of evidence, AD-MSCs have been shown to effectively treat hypertrophic scars by promoting HSF apoptosis and inhibiting their proliferation and migration—an effect that may be related to the inhibition of Nrf2 expression in HSFs (Li et al., 2022). Consistent with these findings, multiple analogous observations have been reported across the literature (Su et al., 2023).

Beyond direct signaling pathway modulation, abnormal metabolic reprogramming plays a crucial role in scar formation throughout the scarring phase of burn wound healing. A key manifestation is an abnormal increase in glycolysis rate (the Warburg effect), which serves as a prerequisite for myofibroblasts to acquire a contractile phenotype and secrete large amounts of collagen. Addressing this metabolic derangement, a diabetic wound healing study demonstrated that MSC-sEVs enhance glucose utilization within wound tissue, thereby accelerating high-quality healing and improving the biomechanical elasticity of regenerated skin (Cai et al., 2023).

In the domain of lipid metabolism, mechanistic evidence further suggests that MSC-sEVs-mediated modulation of lipid metabolic pathways associated with HSF proliferation and ECM synthesis may reduce aberrant scar formation (Cui et al., 2022). Critically, this metabolic dimension of regulation offers a conceptual framework for scar-free healing that extends beyond canonical signal transduction inhibition, representing a paradigm shift from targeting signaling nodes to reprogramming the metabolic circuitry of fibrosis.

### 6.2 Prospective scar management prospective

Prospective scar management, initiated from the wound closure phase, incorporates MSC/EVs-based interventions into a 6–12 months post-burn therapeutic pathway, aiming to achieve early-stage intervention and sustained long-term scar control. The primary focus of this phase is the precise modulation of signaling pathways—including Wnt/β-catenin—by sEVs, with the explicit goal of optimizing ECM architectural organization. Regarding delivery modalities, given that exosomal penetration through intact or remodeling skin surfaces is inherently limited, innovation in delivery systems is directly determinative of translational efficacy. For hypertrophic scars exhibiting marked tissue heterogeneity, intralesional injection is considered a precision delivery strategy, as it ensures high-concentration EVs exposure directly within the HSF-dense core of the lesion. Although current clinical evidence is predominantly derived from small-scale studies or analogical experiments, the excellent biocompatibility and minimal immunogenicity of exosomes confer a safety profile superior to that of glucocorticoids for repeated, long-course intralesional administration (Zhou et al., 2023). Consistent with this rationale, local injection ensures targeted high-concentration exosome delivery to the lesional zone and is particularly suited to hypertrophic scars with pronounced tissue heterogeneity (Li et al., 2021; Xu et al., 2024). Concerning combination with physical modalities such as laser therapy, although an explicit “laser + exosome” combination protocol has not yet been formally described, existing literature indicates that exosomes can function as bioactive scaffold-integrated carriers to promote scar-free healing (Manzoor et al., 2024). Regarding the design of clinical translation and evaluation frameworks, a staged assessment schedule with timepoints at 6, 9, and 12 months constitutes a rational and comprehensive evaluation system. This schedule should evaluate scar height, elasticity, vascularity, pigmentation, and patient-reported outcomes (including pain and cosmetic satisfaction), combined with imaging modalities such as ultrasound and optical coherence tomography and histological parameters including collagen architecture and alpha-smooth muscle actin (α-SMA) expression. Critically, exosome source, culture conditions (2D versus 3D) (Zhu et al., 2024; Camões et al., 2022), isolation and purification methodologies, and quality control standards must be rigorously standardized to ensure therapeutic reproducibility and cross-institutional consistency (Li S. et al., 2024). In addition, carrier biocompatibility and ethics committee approval, particularly where iPSC-derived exosomes are involved (Bo et al., 2022), must also be incorporated into protocol design. Taken together, the construction of a prospective scar management system centered on sEVs, integrating intelligent delivery platforms with multimodal outcome assessment, represents the necessary path forward for burn rehabilitation, transitioning from mere wound coverage toward complete regeneration.

## 7 Product forms and drug delivery platforms for MSC/EVs in burn therapy

### 7.1 Delivery innovation and carrier systems for burn applications

Live MSCs possess the capacity to dynamically sense and respond to the wound microenvironment while continuously secreting a broad repertoire of growth factors and cytokines, providing multidimensional signaling regulation throughout wound healing. However, their clinical translation faces multiple barriers, including low *in vivo* survival rates, potential tumorigenic or immunogenic risks, and complex regulatory approval pathways, as evidenced by recent investigations (Wang et al., 2025a).

The mode of administration directly determines the duration of MSC/EVs exposure within the inflammatory network, their spatial distribution, and their targeting efficacy, thereby influencing both immune reprogramming of the wound microenvironment and systemic inflammatory control. Topical application via spraying or hydrogel-based loading emphasizes achieving high-concentration, controlled cargo exposure at the wound bed level, leveraging sustained-release kinetics and material properties to achieve “*in situ* immunomodulation.”

Topical delivery is generally associated with lower systemic exposure, theoretically reducing risks of systemic immunosuppression, systemic infection, and off-target organ toxicity. Nevertheless, burn wound surfaces frequently present significant barriers to drug penetration due to exudate accumulation and necrotic tissue burden. Studies have noted that existing hydrogel systems often achieve suboptimal therapeutic outcomes owing to their inability to conform to irregular wound topographies or their limited antimicrobial capacity (Yu Q. et al., 2023), representing practical challenges that must be addressed by topical delivery strategies. Selected investigations, such as that by Vipin et al., have developed stimuli-responsive materials (e.g., pH- and enzyme-sensitive hydrogels) to improve exosome release kinetics within complex pathological microenvironments, demonstrating enhanced therapeutic efficacy (Vipin and Kumar, 2025).

In contrast, systemic intravenous infusion provides broad-spectrum exposure, distributing MSC/EVs-mediated signals across multiple nodes of the circulating immune network. This approach is theoretically more advantageous for controlling SIRS and mitigating the risk of multiple organ dysfunction in large TBSA burns. Curtis et al. (2019) demonstrated that intravenous MSC administration attenuates systemic inflammation and reduces hepatic and pulmonary apoptosis and inflammatory injury, suggesting MSCs as a novel therapeutic modality for restoring multi-organ homeostasis in alcohol-complicated burn patients. Intravenous infusion is preferred in clinical trials owing to its technical simplicity, minimal invasiveness, and repeatability. However, due to the “first-pass effect,” intravenously administered MSCs are predominantly sequestered in the pulmonary vasculature and undergo rapid clearance post-injection (Pang et al., 2021). Yeung et al. fabricated MSC spheroids using hanging-drop culture and administered them intravenously in cynomolgus monkeys. Their results demonstrated that MSC spheroid infusion yields superior cell survival, reduced pulmonary entrapment, and enhanced therapeutic efficacy compared with conventional MSC suspension (Yeung et al., 2022), offering a novel avenue for MSC-based therapy.

At the carrier level, advanced technologies including smart hydrogels and three-dimensionally bioprinted scaffolds provide combinatorial platforms offering sustained release, site-specific delivery, and structural support for MSC-sEVs. For instance, Liu et al. (2025b) encapsulated antagomiR-192-5p-loaded MSC-sEVs within gelatin methacryloyl (GelMA) hydrogels modified with MXene (Ti_3_C_2_T_X_) nanosheets to generate a multifunctional wound dressing (Exo-ant-192@M-Gel). This construct achieved sustained anti-miR-192 release, retarded vesicle degradation, exerted anti-inflammatory effects, and promoted wound healing in a rat burn model. This construct achieved sustained anti-miR-192 release, retarded vesicle degradation, exerted anti-inflammatory effects, and promoted wound healing in a rat burn model. Similarly, a novel hydrogel scaffold fabricated from recombinant human collagen type I and carboxymethyl chitosan and loaded with umbilical cord MSC-sEVs extended exosome retention time at the wound site and accelerated wound repair (Wu et al., 2024). To address the challenges of controlling hostile microenvironmental conditions and rapid cellular egress from hydrogel constructs under pathological states, Lee et al. (2025) demonstrated that the synergistic effect of hypoxic conditioning and cell-adhesive colloidal gels enhances MSC paracrine factor productivity and accelerates neovascularization. Microneedle systems represent an effective strategy for transdermal drug delivery. Zhang et al. (2023e) proposed a novel biomimetic adaptive impregnated microneedle platform for diabetic wound healing, fabricated through a combined template-replication and three-dimensional transfer printing manufacturing strategy. This platform consists of tunable polyvinyl alcohol (PVA) hydrogel tips loaded with MSC-sEVs and a detachable 3M medical tape backing substrate, demonstrating effective promotion of tissue regeneration and diabetic wound healing. Similarly, researchers combined 3D cultured UC-MSC-sEVs with methacrylated hyaluronic acid (HAMA) microneedle arrays to form an Exo@HAMA microneedle system (Zhu et al., 2024). This platform enables non-invasive transdermal delivery, precisely delivering exosomes into wound tissue via microneedle penetration, while leveraging the controlled-release characteristics of microneedle systems to sustain long-acting exosome bioavailability, effectively promoting the healing of deep partial-thickness burns.

### 7.2 From laboratory preparation to GMP: quality control and reproducibility

Live MSCs possess the capacity to dynamically respond to the microenvironment and continuously secrete diverse growth factors and cytokines, providing multidimensional signaling regulation throughout wound healing. However, clinical translation confronts multiple barriers, including low *in vivo* survival rates, potential tumorigenic or immunogenic risks, and complex regulatory pathways (Wang et al., 2025a). By contrast, MSC-sEVs offer superior stability, lower immunogenicity, and greater flexibility in storage and transportation, and have emerged in recent years as a prominent candidate for cell-free therapeutics (Tieu et al., 2020). Nevertheless, the efficacy and safety of MSC-sEVs in clinical translation remain subjects of ongoing debate, primarily attributable to four categories of unresolved issues: (1) heterogeneity in MSC manufacturing processes; (2) variability in exosome production and isolation methodologies; (3) absence of standardized quality assurance testing methods; and (4) insufficient reproducibility across *in vitro* and *in vivo* functional studies (Witwer et al., 2019).

Regarding heterogeneity in MSC preparation methods, donor and source consistency constitutes the principal determinant of product stability. MSCs derived from different tissue sources, including bone marrow, adipose tissue, umbilical cord, and burn-excised skin waste, exhibit substantial variation in phenotype, proliferative capacity, immunomodulatory potency, and tissue repair efficacy (Dolp et al., 2021). Even within a single cell line, critical quality attributes (CQAs) change progressively with increasing passage number and under varying freeze-thaw conditions (Lechanteur et al., 2021). More critically, inter-donor variability within the same tissue source cannot be overlooked. The exosomal miRNA profiles, proteomes, and functional activities of MSCs from distinct tissue origins differ substantially, and individual donor-to-donor variation exists even within the same tissue source (Asch et al., 2025). This heterogeneity manifests not only at the cellular level but is directly propagated to the secreted extracellular vesicle fraction. Analysis of serum-derived exosomes from 29 burn patients and 13 healthy controls demonstrated marked inter-batch heterogeneity inherent to clinical samples, indicating that if manufacturing processes are not standardized, batch-to-batch variation will directly confound therapeutic efficacy assessment (Ding et al., 2023). Therefore, unambiguous specification of tissue source at the outset of product development, establishment of source-specific CQA frameworks, and standardization of final product storage protocols with validated acceptance criteria are prerequisites for ensuring product consistency.

Regarding differences in exosome production and isolation technologies, isolation and purification methodology represents a critical process determinant of exosome product quality. The manufacturing approach directly dictates particle size distribution, purity, cargo composition, and biological function (Liangsupree et al., 2021). Current methodologies, including differential ultracentrifugation (dUC), size-exclusion chromatography (SEC), ultrafiltration (UF), tangential flow filtration (TFF), and immunoaffinity capture, each present distinct advantages and limitations (Zheng et al., 2024). Although dUC remains the most widely employed laboratory technique, it is operationally laborious, poorly scalable, and susceptible to co-pelleting of non-vesicular contaminants including lipoproteins and apoptotic bodies, compromising product purity (Bok et al., 2024). SEC and TFF achieve a more favorable balance between purity and yield and demonstrate greater feasibility in GMP-compliant environments. The combined TFF and SEC process has been proven to significantly improve exosome purity and holds promise for achieving large-scale production (Liu et al., 2025c). Notably, particle-to-protein ratios in products obtained by different isolation methods vary considerably, differing by as much as 10- to 100-fold between batches, directly undermining batch reproducibility and rendering cross-study dose comparisons methodologically invalid (Takov et al., 2019). Accordingly, isolation and purification protocols should be finalized and locked early in product development, validated across multiple production runs, and formally incorporated into manufacturing procedures.

The lack of standardized quality assurance testing methods mainly encompasses two aspects: (1) characterization standards and efficacy testing, and (2) standardization of administration targets and dosing.

Characterization standards and potency assays represent core GMP compliance requirements and currently constitute one of the most critical gaps in the field. The MISEV2023 guidelines issued by the International Society for Extracellular Vesicles (ISEV) in 2023 explicitly mandate that EV preparations be characterized by at least two orthogonal particle sizing techniques (e.g., nanoparticle tracking analysis [NTA] combined with transmission electron microscopy [TEM] or cryogenic electron microscopy [cryo-EM]) for concurrent characterization of size distribution and particle concentration. Additionally, positivity rates for tetraspanin markers CD63, CD9, and CD81, as well as residual host cell protein contaminants such as glucose-regulated protein 94 (GRP94), must be assessed to establish product identity and purity (Welsh et al., 2024). However, physicochemical characterization alone is insufficient to support release decisions. In the domain of potency testing, Lei et al. (2025) identified 28 critical miRNAs within MSC-sEVs closely associated with regenerative, anti-inflammatory, and anti-fibrotic functions. They demonstrated that artificially engineered exosome-like vesicles carrying this specific miRNA signature can replicate the burn wound healing and scar-reducing efficacy of native MSC-sEVs, providing an important foundation for establishing miRNA functional profile-based potency assays, similar to the CQAs in the acute inflammatory phase and scar remodeling phase listed in Table 1.

**TABLE 1:** Burn pathophysiology phases and therapeutic evaluation framework.

Pathological phase	Indication	Optimal intervention window	Route of administration	Delivery strategy	Core Quality Attributes (CQA)
Acute inflammatory Phase (0–72 h)	Large TBSA burns (>20%), rescue of wound zone of stasis	Within 24 h post-injury	Systemic intravenous (IV) infusion + topical spray application	Phosphate-buffered saline or neat suspension (direct application)	Anti-inflammatory miRNA; NLR family pyrin domain-containing protein 3 (NLRP3)inhibitory potency
Proliferative phase (4–21 days)	Deep partial-thickness burns; adjunct to skin grafting in full-thickness burns	Intraoperatively or following surgical debridement	Topical application; bioactive dressing-loaded deliverys	sEV-loaded hydrogels; impregnated acellular dermal matrix (ADM)	Topical application; Pro-angiogenic factors (VEGF, FGF); pro-migratory proteins
Remodeling Phase (21 days–1 year)	Contracture-prone anatomical sites; high-risk individuals for hypertrophic scarring	Commencing 1–3 months post-wound closure	Transdermal delivery; intralesional injection	Microneedle patches (MN); laser-assisted transdermal delivery	Anti-fibrotic miRNA, HGF; metabolic reprogramming capacity

Dosing metrics and dose standardization represent a long-standing and unresolved critical gap in translational research. Different clinical trials report MSC-sEVs doses using inconsistent units, including particle number, protein content, RNA quantity, or volume. A systematic review of MSC-sEVs clinical trials conducted between 2014 and 2024 identified the absence of defined dose-response relationships as one of the most overlooked core deficiencies in current clinical translation and proposed the adoption of a dual-metric dosing framework combining particle number with protein content, supported by mechanism-driven *in vitro* potency assays, to provide a biologically meaningful pharmacological basis for dosing (Wang et al., 2025d). Furthermore, current industrial-scale exosome yields rarely exceed 10^13^ particles per liter of culture medium—significantly below therapeutically required levels—and the production capacity bottleneck for large-scale manufacturing remains an urgent challenge (Bagdasarian et al., 2021).

Storage stability and batch consistency are equally essential prerequisites for GMP translation. MSC-sEVs are typically stored at −80 °C in phosphate-buffered saline. However, no harmonized standards exist regarding the cumulative impact of storage duration, freeze-thaw cycling frequency, and cryoprotectant formulation on vesicle functional integrity. MISEV2023 explicitly mandates detailed documentation and reporting of all storage variables and their effects on EV activity (Welsh et al., 2024). For GMP-grade burn therapeutic products, stability studies should establish product shelf-life specifications under pre-defined storage temperatures, with acceptance criteria linked simultaneously to physicochemical identity parameters (particle size and concentration) and functional potency dimensions. Batch consistency must be validated across a defined number of independent manufacturing runs, with statistical acceptance criteria prospectively established within a Quality by Design (QbD) framework (Maillot et al., 2021).

At the regulatory and industrialization level, as of 2025, no MSC-sEVs product has received marketing authorization from the Food and Drug Administration (FDA), European Medicines Agency (EMA), or equivalent regulatory authorities worldwide, reflecting the fragmented global regulatory landscape with respect to EVs product classification, GMP compliance requirements, and clinical evaluation standards (Wang et al., 2025d). In China, the Quality Control Standards for Mesenchymal Stem Cell Exosomes (T/SBIAORG 001-2023) published by the Shanghai Biopharmaceutical Industry Association in 2023, and the Standards for the Preparation and Testing of Human-Derived Mesenchymal Stem Cell Exosomes published by the China Food and Drug Enterprise Quality and Safety Promotion Association in 2024, provide systematic guidance covering cellular raw materials, manufacturing processes, and quality standards, constituting important reference frameworks for product development.

In summary, the construction of a standardized system for MSC-sEVs burn therapeutic products, spanning donor consistency, isolation and purification, characterization standards, potency assays, dosing norms, storage stability, and regulatory compliance, constitutes a comprehensive systems-level undertaking. Only through the establishment of unified, standardized manufacturing and quality control frameworks can the reproducibility of multi-center research be fundamentally improved, enabling the accumulation of high-quality clinical evidence and ultimately achieving true translational advancement of MSC-sEVs from bench to bedside.

## 8 Clinical translation and future directions

### 8.1 Animal models

Many studies have already explored cell therapies for burn healing in animal models. Yi et al. (2020) found that across 20 studies of MSC treatment for burn wounds, cell therapy improved wound closure rates (2.00, 95% CI: 0.52–3.48, p = 0.008) and reduced wound area (−2.36; 95% CI: −4.90 to 0.18; p = 0.069); moreover, MSCs significantly improved vascular density (4.69; 95% CI: 0.06–9.31; p = 0.047) and crust thickness (6.56, 95% CI: 1.15–11.98, p = 0.017). Current data on stem cell use for burn wound healing in animal models are summarized in Table 2.

**TABLE 2:** Partial animal-model validation of MSC/Exosome therapy for burns.

Stem cell type	Model	Route of administration	Mechanism of action	References
BM-MSCs	Rats	Subcutaneous injection	Improves deeper second-degree burn healing by downregulating IL-6, TNF-α, TGF-β, MMP-9 and miR-21 expression; significantly upregulates HSP90α in later healing	Abdel-Gawad et al. (2021)
BM-MSCs	Rats	Tail vein injection after Fe3O4 NP labeling	Increases CD31 and α-SMA levels; reduces IL-1α, IL-2, IL-6 and IFN-γ	Li et al. (2020)
BM-MSCs	Rats	Subcutaneous injection	Stimulates Akt/mTOR signaling to improve healing	Ramhormozi et al. (2020)
BM-MSCs	Rats	Subcutaneous injection	Overexpression of Caveolin-1 increases TGF-β1, TGF-β3, FGF and EGF; decreases IL-1β, IL-6, TNF-α; accelerates burn wound healing	Wu Z. et al. (2021)
BM-MSCs	Rabbits	Conjunctival/subconjunctival injection	Reduces tissue inflammation, enhances corneal repair and stimulates cell turnover to promote alkali burn healing	Hassan et al. (2025)
UC-MSCs	Rats	Tail vein injection	Regulates liver P38 MAPK and NF-κB P65 phosphorylation to reduce HMGB-1, IL-6, TNF-α and increase anti-inflammatory IL-10, alleviating inflammation	Jian-Xing et al. (2021)
UC-MSCs	Rats	Subcutaneous injection	Increases CD31 and VEGF in ulcers, promoting neovascularization; related to the PI3K/Akt pathway	Liu et al. (2018)
UC-MSCs	Mice	Tail vein injection	Reduces blood–brain barrier permeability to decrease serum and brain IL-6 and IL-1β, thereby reducing inflammation	Yang et al. (2020)
UC-MSCs	Baima miniature pig	Intradermal injection	Promotes wound epithelialization, reduces wound inflammatory responses including inflammatory cell infiltration and cytokines, increases microvasculature, accelerating healing	Liu L. et al. (2024)
UC-MSCs	Mice	Wound-local exosome patch	Regulates fibroblasts, endothelial cells, and macrophages; inhibits myofibroblast-driven fibrosis; promotes tissue regeneration and inhibits scar formation	Yang et al. (2024)
hAMSCs	Mice	AMSCs + AMSCs-CM; subcutaneous injection	Activates PI3K/AKT signaling; *in vitro* strongly inhibits heat-stress–induced apoptosis in HaCaT and DFL cells; promotes proliferation via GSK3β/β-catenin	Li et al. (2019)
hAMSCs + AD-MSCs	Rats	Decellularized hAM (DhAM) combined with AD-MSCs	Releases high concentrations of adenosine (CD73) to modulate inflammation	Naasani et al. (2022)
hAMSCs	Rabbits	Anterior chamber injection	Secretes soluble factors (e.g., TSG-6) to inhibit activated neutrophil NET release, reduce neovascularization, edema, infiltrating inflammatory cells, and α-SMA^+^ cells	Navas et al. (2018)
PMSCs	Mice	Subcutaneous injection	Overexpression of IGF-1 reduces proinflammatory IL-1β, IL-6, TNF-α; lowers TGF-β1, collagen I and III expression; increases VEGF to inhibit inflammation and collagen deposition, accelerating healing	Cheng et al. (2021)
PMSCs	Rats	Wound topical dressing	Accelerates migration and proliferation of dermal fibroblasts and keratinocytes to promote regeneration, stimulates angiogenesis, reduces scarring; secretes PGE2, IL-6, IL-8 or IFN-γ, IL-10, growth factors and chemokines that influence macrophages and neutrophils to mitigate *S. aureus* burns (II–IIIa)	Kudinov et al. (2021)
PMSCs	Rats	Conjunctival/subconj injection	Inhibits inflammatory cytokines IL-1β, MCP-1 and MMP9; polarizes CD206+ M2 macrophages, effectively promoting corneal alkali burn repair	Chen M. et al. (2023)
AD-MSCs	Sheep	Wound topical application	Increases VEGF expression to enhance blood flow	Fujiwara et al. (2020)
AD-MSCs	Rats	Wound topical application	Reduces duration of the inflammatory phase, improves re-epithelialization, and increases collagen deposition	Hermeto et al. (2020)
AD-MSCs	Rats	Wound topical dressing (AD-MSCs + composite)	Significantly reduces inflammation, increases collagen deposition, improves vascularization, accelerating wound closure	Rasouli et al. (2024)
AD-MSCs	Rats	Wound topical dressing (AD-MSCs + PCL/gel nanofibers)	Reduces IL-6 and IL-1β, alleviates inflammation, increases glycosaminoglycans and hydroxyproline content, reduces α-SMA, preventing fibrosis	Shahmohammadi et al. (2024)
AD-MSCs	Mice	Gel-treated local delivery	Improves wound re-epithelialization, increases vascular distribution, upregulates pro-angiogenic MCP-1, VEGF, and SDF-1 at mRNA and protein levels; downregulates anti-fibrotic TIMP1 and pro-inflammatory TNFα	Barrera et al. (2021)
AD-MSCs	Mice (third-degree burns)	Wound topical dressing	Differentiates into fibroblasts and keratinocytes, promotes wound healing; stimulates vascular tissue formation, secretes growth factors, synthesizes new ECM and modulates other cells	Cabello-Arista et al. (2024)
AD-MSCs	Rats	Peri-wound subcutaneous injection	Increases TGF-β and VEGF gene expression, increases fibroblast and vascular numbers, improves collagen deposition, reduces wound inflammation	Rezaei Yazdi et al. (2021)
AD-MSCs	Rats	Peri-wound subcutaneous injection	Increases type III collagen deposition, decreases lymphatic vessel numbers, improves wound healing	Franck et al. (2019)
AD-MSCs	Rats	Intradermal injection	Reduces wound inflammation by providing immunomodulation in the early burn phase; modulates microenvironment to promote healing over large-area burns	Gürünlüoğlu et al. (2024)
AD-MSCs	Rabbits	Conjunctival injection	Reduces tissue inflammation, enhances corneal repair, stimulates cell turnover to promote alkali burn healing	Hassan et al. (2025)
AD-MSCs	Mice, dogs	Peri-wound subcutaneous injection	Positively modulates epidermal repair, collagen deposition, hair follicle vascularization, and inflammatory response; significantly shortens wound healing time; beneficial for diabetic skin injuries in dogs	Kou et al. (2023)
Induced mesenchymal stem cells (iMSCs)	Pigs	iMSCs incorporated into mature epidermal–dermal substitutes	Lowers inflammatory factor levels, improves Type I/III collagen ratio, promotes VEGF expression and capillary neogenesis to accelerate burn wound healing	Farahat et al. (2025)

In summary, the data presented in Table 2 indicate that subcutaneous or peri-wound injection represents the most effective administration strategy across diverse species and MSC origins—a finding corroborated by Wang et al. (2024). In a direct comparison of local wound dressing, peri-subcutaneous injection, and tail vein injection in a mouse full-thickness skin defect model, peri-subcutaneous injection yielded the strongest therapeutic effect. This observation suggests that administration route selection constitutes a key variable requiring standardization in future clinical protocols. Regarding mechanistic outcomes, anti-inflammatory and pro-angiogenic effects emerge as the most consistently reported results across studies. Specifically, in investigations involving BM-MSCs, UC-MSCs, and AD-MSCs, IL-1β, IL-6, and TNF-α levels were consistently decreased, whereas CD31 and VEGF expression was correspondingly increased. This convergent pattern suggests that these mechanisms represent core, origin-independent therapeutic effects. By contrast, the anti-fibrotic effect displays notable inconsistency. Although most studies interpret reduced α-SMA and collagen deposition as beneficial outcomes, Rezaei Yazdi et al. (2021) and Franck et al. (2019) have interpreted increased collagen deposition as evidence of improved structural scaffold formation. This discrepancy underscores that collagen-related endpoints are inherently context-dependent and cannot be uniformly regarded as surrogate markers of therapeutic benefit.

A fundamental limitation that constrains the translational value of these animal data lies in the substantial biological differences between human and animal skin. Wounds in mice and rats heal primarily through contraction rather than reepithelialization, yielding higher wound closure indices that may not accurately reflect the reepithelialization-dependent healing that predominates in human burns. Compounding this issue, inflammatory responses in animals subside more rapidly and predictably than in humans. By contrast, chronic inflammation, persistent immune dysregulation, and hypertrophic scarring constitute major complications in the human clinical context. Driven by these pathological discrepancies, the use of porcine models—as employed by Liu L. et al. (2024) and Farahat et al. (2025) may provide a closer approximation to human skin anatomy, burn depth pathology, and healing dynamics.

### 8.2 Clinical evidence; trials and approved products

MSC-based burn wound repair therapies have entered the clinical evaluation stage, encompassing various cell sources and administration strategies. Relevant clinical trials and case reports are detailed in Table 3. Currently, mesenchymal stem cell-based burn wound repair therapies remain predominantly in the clinical trial stage; although a few therapies have obtained Investigational New Drug (IND) approvals, no marketed products are yet available (Table 4).

**TABLE 3:** Some clinical trials and case reports of MSCs in burn treatment.

Study	Indication	Treatment modality	Measured outcomes/therapeutic effects	Phase	Number/References
BM-MSCs	Eye burns, grade IV or higher	5 × 10^6^/0.5 mL MSCs injected into the inferior fornix; persistent epithelial defect required a second treatment	At 6 months: incidence of corneal perforation after subconjunctival injection, corneal epithelialization time, and vision	II	NCT02325843
UC-MSCs	Acute, moderate to severe full-thickness burns	Standard care + UC-MSCs	At 6 months: assessment of related skin parameters	I/II	NCT01443689
UC-MSCs	Eye chemical/thermal burns	0.2 mL (∼2 × 10^6^ cells) subconjunctival injection	At 3 months: evaluation of corneal perforation percentage	I/II	NCT03237442
UC-MSCs	Burns involving >30% TBSA	Two groups: external group with hyaluronic acid gel carrier; subcutaneous injection group	Healing time, scar score, and scar tissue immunohistochemistry as primary endpoints	Exploratory/Preliminary	ChiCTR2000040932
MSCs	Superficial second-degree burns (4 groups, 5 per group)	Graded doses: initial 2.5 × 10^3^/cm^2^; second 5 × 10^3^/cm^2^; third 1 × 10^4^/cm^2^; fourth 2 × 10^4^/cm^2^	Monitoring adverse events after allogeneic MSC administration (CTCAE v4.0) over 1.5 years	I	NCT02104713
AD-MSCs	Deep second-degree burns ≥100 cm^2^	ALLO-ASC-DFU (hydrogel film containing allogeneic adipose-derived MSCs)	Within 4 weeks: number of adverse events as safety and tolerability measure	I	NCT02394873
AD-MSCs	Second- to third-degree burns with wounds	Allogeneic adipose-derived MSCs + platelet-poor plasma fibrin hydrogel tissue construct	Within 1 month: degree of flap healing after autograft	I/II	NCT03113747
AD-MSCs	Deep second-degree burn wounds ≥100 cm^2^; TBSA ≤30%	Hydrogel film containing allogeneic adipose-derived MSCs	12-week follow-up re-epithelialization time	II	NCT02619851
BM-MSCs	10 adults with deep second-degree burns	Five patients receive 2.5 × 10^3^ BM-MSC/cm^2^, while the other five receive 5 × 10^3^ allogeneic BM-MSC/cm^2^	All patients responded well; wounds 100% closed	Completed	Schulman et al. (2022)
UC-MSCs	90 primiparous women undergoing elective cesarean	Randomized to placebo, low-dose (3 × 10^6^ cells) or high-dose (6 × 10^6^ cells) percutaneous hydrogel UC-MSCs	At 6 months, mean total VSS scores: placebo 6.43, low-dose 5.18, high-dose 4.71	Completed	Fan et al. (2020)
hAMSCs	One 65-year-old patient with extensive burns covering 26% TBSA	Allogeneic amniotic membrane–derived MSC transplantation + combination therapy	Discharged on day 77; skin parameters gradually improved	Completed	Czerny-Bednarczyk et al. (2025)
BM-MSCs	16 acute severe ocular burns (Dua’s grade 4–VI)	Subconjunctival injection alongside standard treatment	13 eyes (81.3%) achieved complete corneal epithelialization by weeks 4–10; effectiveness 87.5% (95% CI: 61.7–98.4)	Completed	Liang et al. (2021)
AD-MSCs	A 25-year-old woman with approximately 65% TBSA deep full-thickness burns	Autologous AD-MSCs seeded at 1 × 10^4^/cm^2^ on Integra® DRT fragment, combined with a collagen scaffold	By week 4, testing area and reference area underwent staged grafting with NHK suspension; at final surgery 9 months later, the boundary between Integra® DRT and unreconstructed skin was indistinct	Completed	Piejko et al. (2020)

**TABLE 4:** Products in the IND stage.

Drug name	Indication	IND number	Approval stage
Human Umbilical Cord–Derived Mesenchymal Stem Cell Injection	Intravenous injection for second-degree burns	CXSL2200301	IND
HS002 Human Umbilical Cord–Derived Mesenchymal Stem Cells	For burn repair, diabetic foot ulcers, and pressure ulcer wounds	CXSL2300424	IND

The clinical evidence summarized in Table 3 indicates that existing data support consistent short-term safety across MSCs from different sources and routes of administration. Specifically, Schulman et al. (Schulman et al., 2022) demonstrated in a phase I dose-escalation trial that 100% wound healing was achieved at all dose levels (2.5 × 10^3^ to 5 × 10^3^ BM-MSCs/cm^2^) without serious adverse events. Complementing these findings, Fan et al. (2020) reported in a randomized controlled trial that patients who received hydrogels containing up to 6 × 10^6^ UC-MSCs percutaneously had significantly lower Vancouver scar scores at 6 months than the placebo group without treatment-related grade ≥3 adverse events. Extending this evidence to severe burn scenarios, a case report of hAMSCs applied to 26% TBSA burns (Czerny-Bednarczyk et al., 2025) and another of AD-MSCs employed for 65% TBSA deep full-thickness burns (Piejko et al., 2020) further demonstrate the feasibility of MSC-based treatments in severe burns. Nevertheless, the case-specific nature of these reports limits the generalizability of their findings. Regarding efficacy endpoints, reepithelialization time and scar assessment scores (VSS, POSAS) are the most frequently reported outcomes, with improvements consistently observed across different MSC sources. This convergence suggests a degree of cross-origin consistency in their core pro-healing and anti-scarring effects. Despite these encouraging observations, the clinical evidence base is subject to several important limitations. A primary concern is that all included trials were Phase I or early Phase I/II, with sample sizes ranging from 1 to 90 participants. Most employed single-center, open-label designs, resulting in a low overall level of evidence. An additional limitation is that none of the trials stratified patients according to burn depth, total body surface area (TBSA), burn location, or inhalation injury—all of which are major determinants of burn prognosis. Consequently, it remains impossible to determine which patient subgroups are most likely to benefit from MSC therapy. Finally, follow-up periods rarely exceeded 6 months, and no trials have incorporated long-term functional endpoints such as range of motion, skin elasticity, pain or pruritus scores, or patient-reported quality of life. As a result, the actual efficacy of MSC therapy in burn rehabilitation remains incompletely characterized. Taken together, although existing clinical data demonstrate objective efficacy and a favorable safety profile for MSC therapy, there remains an urgent need to conduct high-quality, multicenter, randomized controlled trials to establish MSC therapy as a standardized component of burn treatment.

### 8.3 Limitations and evidence gaps

Current clinical and translational research on MSC/EVs in burn treatment remains in an early exploratory stage. Although the existing literature consistently indicates that MSC/EVs demonstrate significant potential in promoting wound healing, regulating inflammatory responses, improving angiogenesis, and reducing scar formation, it is equally evident that the relevant clinical evidence is subject to multiple critical limitations.

A prominent challenge is the high degree of heterogeneity in cell sources, and a lack of systematic comparative studies. Existing studies have included MSC sources from burn skin waste tissue (BD-MSCs) (Dolp et al., 2021), adipose tissue (AD-MSCs) (Schumacher et al., 2024), and bone marrow (BM-MSCs) (Wang et al., 2020). Critically, cells from different sources differ substantially in their biological characteristics, secretory profiles, and therapeutic effects. Despite this recognized variability, there remains a conspicuous absence of systematic, targeted comparative studies specifically for burn indications, and no consensus has been reached regarding the optimal MSC source. Supporting this concern, a systematic review and meta-analysis of 83 studies confirmed that MSC-sEVs exert a clear therapeutic effect on wound healing and skin regeneration. However, subgroup analysis further revealed that differences in EV source, vesicle subtype, and preparation method significantly influenced efficacy outcomes, underscoring that source specificity constitutes a key determinant of efficacy reproducibility (Almeida et al., 2025).

An equally critical issue is the absence of unified standards for exosome preparation and quality control. There are currently no unified specifications for exosome isolation methods (differential ultracentrifugation, SEC, TFF, etc.), characterization standards (NTA, TEM, surface markers), potency testing protocols, and dosage indicators. Driven by these inconsistencies, the variability in dose reporting units across trials—including particle number, protein content, RNA quantity, or volume—renders the establishment of dose-response relationships virtually impossible (Wang et al., 2025d) Compounding this problem, most existing studies have not adhered to the minimum characterization requirements outlined in MISEV2023, and batch-to-batch variation remains unvalidated, thereby hindering the reproducibility of multicenter studies and regulatory approval processes. In line with these observations, a review that systematically integrated the efficacy of MSC-sEVs across various disease models similarly identified the lack of standardization as the primary obstacle to clinical translation, calling on researchers to implement MISEV2023-compliant reporting (Mussin et al., 2025).

A further limitation pertains to the weakness of clinical trial design and the scarcity of long-term outcome data. Existing burn-related clinical trials generally suffer from design deficiencies, including small sample sizes, single-center settings, lack of blinding, and short follow-up periods. Consequently, outcome indicators predominantly focus on short-term endpoints such as wound closure time, while systematic evaluation of patient-relevant outcomes—including long-term functional recovery, scar elasticity, vascularization quality, pain, and pruritus—remains lacking (Santuzzi et al., 2024). Corroborating this assessment, a recent meta-analysis on MSC efficacy in second-degree burn wound repair similarly noted that the quality of existing evidence is uneven, with most studies having follow-up periods of less than 6 months, thereby failing to reflect true long-term prognosis (Schulman et al., 2025). Moreover, subgroup analyses based on burn characteristics—including depth, TBSA, anatomic location, and inhalation injury—are exceedingly rare. As a result, it remains impossible to identify the patient populations most likely to benefit (Tedesco et al., 2025), which in turn impedes the development of precise, stratified intervention strategies. Beyond the limitations discussed above, current research also suffers from a lack of optimization in drug delivery strategies. Currently, much of the evidence for MSC-sEVs in burns remains at the animal model level, primarily focusing on rodents. The complexity of the human skin microenvironment (such as damage to different zones and systemic inflammatory responses) is difficult to fully simulate with simple models. Consequently, key parameters such as administration timing, route of delivery, carrier material-EV compatibility, and release kinetics remain unsupported by clinically relevant optimization data (Zhao H. et al., 2023).

A final critical dimension involves the need for further improvement in GMP production and clinical endpoint design. To advance MSC/EVs therapy from the laboratory to the clinical setting, standardized production processes need to be established within the GMP framework and the Quality by Design (QbD) system (Maillot et al., 2021). Currently, two human umbilical cord-derived MSC injections have received domestic IND approval; however, no GMP-grade MSC-sEVs burn products have completed registration applications. From a trial design perspective, the primary endpoint should extend beyond wound closure to incorporate comprehensive indicators, including scar quality (Vancouver Scar Scale [VSS]; Patient and Observer Scar Assessment Scale [POSAS] score), skin elasticity, vascular density, functional activity, and patient-reported outcomes (pain, pruritus, and aesthetic satisfaction). To address these requirements, minimum methodological standards for multicenter randomized controlled trials should be established to promote the generation of high-quality clinical evidence.

In summary, MSCs and their EVs demonstrate broad biological potential in burn repair. Nevertheless, to achieve standardized, individualized, and efficient clinical application, systematic improvements are needed simultaneously across multiple dimensions, including terminology standardization, source comparison, preparation standards, trial design, outcome measurement, and patient stratification.

## 9 Summary and outlook

In biological and translational research on burn repair, MSC/EVs demonstrate substantial multi-target regulatory potential. Critically, their effects are not confined to a single signaling pathway but instead span the entire dynamic disease course from the acute inflammatory phase through the proliferative phase and into the remodeling phase. During the acute inflammatory phase, MSC/EVs can suppress excessive inflammatory amplification through immuno-inflammatory reprogramming, alleviate endothelial activation and permeability imbalance, and, to a certain extent, limit inflammation-related processes such as neutrophil extracellular trap (NET) formation. Collectively, these actions contribute to generating a modulable microenvironment that is conducive to subsequent repair. In the proliferative phase, encompassing re-epithelialization and angiogenesis, EVs accelerate wound healing through multiple complementary mechanisms, including promoting functional angiogenesis, supporting keratinocyte migration and adhesion, regulating fibroblast behavior, and driving metabolic reprogramming. Upon entering the remodeling phase, MSC/EVs facilitate long-term regulation of the fibrotic network at the levels of anti-fibrosis and extracellular matrix (ECM) remodeling, thereby offering novel strategies for preventing hypertrophic scarring and optimizing scar quality.

It is important to emphasize that the applicability of mechanistic evidence differs substantially across levels of analysis. In burn-specific preclinical studies, MSC/EVs demonstrate more consistent, multidimensional regulation of the inflammatory response. By contrast, evidence derived from upstream pathways frequently originates from similar injury models or *in vitro* systems. Driven by this inherent disparity, the burn-specific applicability of such findings must be rigorously validated using burn-specific models and mechanistic endpoints. Accordingly, future research should adopt an evidence-grading strategy at the mechanistic level, distinguishing between “specificity studies” and “similarity studies” and thereby avoiding the conflation of indirect inference with direct evidence.

Although mechanistic progress provides a solid foundation for translation, current clinical and translational evidence continues to face multiple limitations. Existing studies generally focus on safety and short-term efficacy; sample sizes are limited, study designs are predominantly small-scale or early-phase, and many fail to rigorously stratify key clinical heterogeneity factors such as burn depth, total body surface area (TBSA), anatomic site, and inhalation injury. Compounding this issue, long-term follow-up and functional outcomes—including skin elasticity, blood supply quality, pain and pruritus, range of motion, and patient-reported outcomes—remain insufficiently characterized. On the manufacturing and regulatory front, there is still no unified standard governing MSC/EVs source selection, manufacturing and purification processes, critical quality attributes (CQAs), and efficacy testing methodologies. Consequently, consensus regarding the dose-response relationship and the optimal delivery timing and delivery platform has yet to be established.

Future key directions may be structured around the logic of “burn staging—mechanistic mainline—delivery window.” On one hand, it is necessary to systematically verify in burn-specific models whether MSC-sEVs can modulate HSF and myofibroblast activation, inflammation-fibrosis coupling, and vascular microenvironment remodeling, while distinguishing burn-specific mechanisms from context-dependent mechanisms. On the other hand, efforts should focus on optimizing administration timing and dosage and establishing standardized EV manufacturing and quality control systems. Driven by these parallel priorities, such advances should ultimately support multicenter, staged clinical trials, complemented by biomarker-based evaluation of efficacy and safety.

## Glossary

11β-HSD1: 11β-hydroxysteroid dehydrogenase type 1
AD-MSCs: Adipose-derived mesenchymal stem cells
ADM: Acellular dermal matrix
AKT: Serine/Threonine Kinase/Protein Kinase B
ANG-2: Angiopoietin-2
ARDS: Acute respiratory distress syndrome
Arg1: Arginase 1
BBB: Blood-brain barrier
BD-MSCs: Burn-derived mesenchymal stem cells
CQA: Critical Quality Attribute
cryo-EM: Cryogenic electron microscopy
DAMPs: Damage-associated molecular patterns
DETCs: Dendritic epidermal T cells
DHT: Dihydrotestosterone
dUC: Differential ultracentrifugation
ECM: Extracellular matrix
EGFR: Epidermal Growth Factor Receptor
EMA: European Medicines Agency
EMT: Epithelial-Mesenchymal Transition
EndMT: Endothelial-to-mesenchymal transition
EPCR: Endothelial protein C receptor
EpSCs: Epidermal stem cells
ERK: Extracellular signal-Regulated Kinase
ESCs: Embryonic stem cells
EVs: Extracellular vesicles
FGF: Fibroblast growth factor
FoxO1: Forkhead Box Protein O1
FVIII: Coagulation factor VIII
GelMA: Gelatin methacryloyl
GMP: Good Manufacturing Practice
GRP94: Glucose-regulated protein 94
HAMA: Methacrylated hyaluronic acid
hAMSCs: Human amniotic membrane-derived MSCs
HGF: Hepatocyte growth factor
HLA-II: Human Leukocyte Antigen Class II
HMGB1: High mobility group box 1 protein
HSFs: Human skin fibroblasts
HSP-90α: Heat shock protein-90α
HSPG2: Heparan Sulfate Proteoglycan 2
IGF-1: Insulin-like growth factor 1
IND: Investigational New Drug
iNOS: Inducible nitric oxide synthase
iPSCs: Induced pluripotent stem cells
ISCT: International Society for Cell and Gene Therapy
ISEV: International Society for Extracellular Vesicles
KCs: Keratinocytes
MAPK: Mitogen-Activated Protein Kinase
MFSD2A: Major Facilitator Superfamily Domain-Containing Protein 2A
MISEV: Minimal Information for Studies of Extracellular Vesicles
miRNAs: MicroRNAs
MMPs: Matrix Metalloproteinases
MODS: Multiple organ dysfunction syndrome
MSCs: Mesenchymal stem cells
MSC-sEVs: MSC-derived small extracellular vesicles
NETs: Neutrophil extracellular traps
NF-κB: Nuclear factor kappa-light-chain-enhancer of activated B cells
NLRP3: NLR family pyrin domain-containing protein 3
NOX4: NADPH Oxidase 4
Nrf2: Nuclear Factor Erythroid 2-Related Factor 2
NSAIDs: Non-steroidal anti-inflammatory drugs
NTA: Nanoparticle tracking analysis
OXPHOS: Oxidative phosphorylation
PDGF: Platelet-derived growth factor
PD-L1/2: Programmed death-ligand 1/2
PGE2: Prostaglandin E2
PI3K: Phosphoinositide 3-kinase
PMSCs: Placenta-derived MSCs
POSAS: Patient and Observer Scar Assessment Scale
PTEN: Phosphatase and Tensin Homolog
PVA: Polyvinyl alcohol
QbD: Quality by Design
ROS: Reactive oxygen species
SEC: Size-exclusion chromatography
sEVs: Small extracellular vesicles
SIRS: Systemic inflammatory response syndrome
STAT6: Signal transducer and activator of transcription 6
STSG: Split-thickness skin grafting
TBSA: Total body surface area
TEM: Transmission electron microscopy
TF: Tissue factor
TFF: Tangential flow filtration
TGF-β: Transforming Growth Factor-β
TIMPs: Tissue inhibitors of metalloproteinases
TNF-α: Tumor Necrosis Factor-α
TNFSF-13: Tumor Necrosis Factor Superfamily Member 13
TSG-6: Tumor necrosis factor-inducible gene 6 protein
UC-MSCs: Umbilical cord-derived mesenchymal stem cells
UF: Ultrafiltration
VCAM-1: Vascular cell adhesion molecule-1
VEGF: Vascular Endothelial Growth Factor
VSS: Vancouver Scar Scale
vWF: von Willebrand factor
α-SMA: Alpha-smooth muscle actin
3D: Three-dimensional
